# A split intein and split luciferase-coupled system for detecting protein-protein interactions

**DOI:** 10.1038/s44320-024-00081-2

**Published:** 2024-12-12

**Authors:** Zhong Yao, Jiyoon Kim, Betty Geng, Jinkun Chen, Victoria Wong, Anna Lyakisheva, Jamie Snider, Marina Rudan Dimlić, Sanda Raić, Igor Stagljar

**Affiliations:** 1 Donnelly Centre, University of Toronto, Toronto, ON Canada; 2 Mediterranean Institute for Life Sciences, University of Split School of Medicine, Split, Croatia; 3 Department of Biochemistry, University of Toronto, Toronto, ON Canada; 4 Department of Molecular Genetics, University of Toronto, Toronto, ON Canada

**Keywords:** Protein-protein Interaction, Tri-part Nanoluciferase, Split Intein, Inhibitor, Drug Discovery, Biotechnology & Synthetic Biology, Pharmacology & Drug Discovery

## Abstract

Elucidation of protein-protein interactions (PPIs) represents one of the most important methods in biomedical research. Recently, PPIs have started to be exploited for drug discovery purposes and have thus attracted much attention from both the academic and pharmaceutical sectors. We previously developed a sensitive method, Split Intein-Mediated Protein Ligation (SIMPL), for detecting binary PPIs via irreversible splicing of the interacting proteins being investigated. Here, we incorporated tripart nanoluciferase (tNLuc) into the system, providing a luminescence signal which, in conjunction with homogenous liquid phase operation, improves the quantifiability and operability of the assay. Using a reference PPI set, we demonstrated an improvement in both sensitivity and specificity over the original SIMPL assay. Moreover, we designed the new SIMPL-tNLuc (‘SIMPL2’) platform with an inherent modularity allowing for flexible measurement of molecular modulators of target PPIs, including inhibitors, molecular glues and PROTACs. Our results demonstrate that SIMPL2 is a sensitive, cost- and labor-effective tool suitable for high-throughput screening (HTS) in both PPI mapping and drug discovery applications.

## Synopsis

The split intein-mediated protein ligation (SIMPL) system for detecting protein-protein interactions has been enhanced by incorporating tri-part Nanoluciferase. This updated system, called SIMPL2, demonstrates significant improvements in performance.

- The incorporation of split luciferase significantly improves the operability and quantifiability of the assay.
- Data processing can improve the sensitivity and specificity of SIMPL2 assay, as suggested by an analysis on reference sets.
- SIMPL2 is applicable to characterizing PPI modulators such as inhibitors, molecular glues and PROTACs.

## Abstract (web summary)

The split intein-mediated protein ligation (SIMPL) system for detecting protein-protein interactions has been enhanced by incorporating tri-part Nanoluciferase. This updated system, called SIMPL2, demonstrates significant improvements in performance.

## Graphical abstract

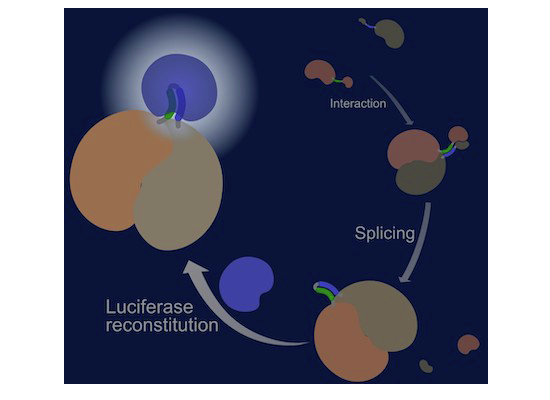

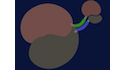

## Introduction

Protein-protein interactions (PPIs) are fundamental biochemical steps in cellular processes (Yao et al, 2017; Snider et al, 2015; Rizzolo et al, 2017; Lopes et al, 2015; Benleulmi-Chaachoua et al, 2016; Lim et al, 2022). Their alterations are involved in various diseases such as cancer, making PPIs attractive therapeutic targets (Saraon et al, 2020; Pathmanathan et al, 2022). However, it is notoriously difficult to target PPIs directly due to the common characteristics of interacting surfaces, which are usually large and flat. Accordingly, PPIs were long considered as “undruggable”. This scenario has been subject to change, however, due to an evolving understanding of protein interaction interfaces and the recent development of new targeting strategies (Arkin and Wells, 2004; Arkin et al, 2014; Scott et al, 2016). Indeed, the milestone PPI inhibitor, venetoclax (Roberts et al, 2017), which targets the BCL2/BAX interaction, has been approved by the FDA for treating chronic lymphocytic leukemia, small lymphocytic leukemia, and acute myeloid leukemia. Numerous PPI inhibitors are also currently under development, with many undergoing clinical trials (Lu et al, 2020). In line with inhibitors, therapeutic potential was also demonstrated for other types of small molecule PPI modulators such as molecular glues that rewire cellular functions through artificially enhancing or enforcing target PPIs (Schreiber, 2021), Proteolysis Targeting Chimeras (PROTACs) that direct targets to the ubiquitin proteasome system for degradation (Neklesa et al, 2017; Pettersson and Crews, 2019), or other chemicals operating through the modality of enhanced proximity (Liu and Ciulli, 2023).

Comprehensive examination of PPIs, both in-depth mechanistic investigation and proteome-scale exploration, is essential for driving biological understanding and drug discovery. However, PPI research heavily depends on technology development. Although numerous techniques for the study of PPIs are available, each one is accompanied by certain limitations (Yao et al, 2015; Snider et al, 2015). Moreover, many methods, whether biophysical or biochemical, are not ideal for applications in PPI-targeted drug discovery. Thus, continued technology advancement is needed to maximize our capabilities in exploring PPIs. To meet this demand, we developed a method called **S**plit-**I**ntein **M**edicated **P**rotein **L**igation (SIMPL) (Yao et al, 2020). For this, we first engineered the GP41-1 split intein and created a version with markedly reduced intrinsic affinity between its two fragments - N-terminal intein (IN) and C-terminal intein (IC)—but without deterioration of its intein activity. Next, we fused the engineered IN and IC, along with two different peptide tags, respectively to two proteins of interest (which we refer to generally as ‘B’ and ‘P’ for convenience). If a PPI occurs between them, spatial proximity allows the IN and IC to reconstitute into a functional enzyme, which splices the B and P proteins into an intact polypeptide or transfers an associated tag from one protein to its partner (depending on the configuration of the constructs). SIMPL demonstrates marked sensitivity and specificity and can be applied to different cellular compartments and various organisms. As part of our initial development of the technology, we demonstrated that SIMPL is compatible with high-throughput screening (HTS) via coupling to the enzyme-linked immunosorbent assay (ELISA). This SIMPL-ELISA platform can also be used for characterizing PPI inhibitors. However, the cumbersome procedures involved in ELISA and the associated expense do not make SIMPL-ELISA an ideal choice for use as an HTS assay. Alternatively, SIMPL can be coupled with homogeneous time-resolved fluorescence (HTRF) (Grozavu et al, 2022), a platform with significantly improved operability. Unfortunately, the cost of reagents and equipment for HTRF limits its widespread application.

Here, we report the incorporation of tri-part Nanoluciferase (tNLuc) (Ohmuro-Matsuyama and Ueda, 2018; Dixon et al, 2017) into the SIMPL system. This new SIMPL-tNLuc (‘SIMPL2’) platform allows detection of splicing in a homogeneous liquid phase, thereby simplifying sample manipulation and markedly improving assay quantifiability. By testing sets of reference PPIs, we show that the tNLuc readout improves sensitivity and specificity. Moreover, we also demonstrate how the modularity of the cell-based SIMPL2 assay platform makes it a valuable tool for characterizing PPI modulators, exemplified with several known small molecule inhibitors, molecular glues and PROTACs. When compared with the NanoBiT assay, we found that SIMPL2 is more sensitive in detecting weak PPIs or induced proximity interactions. Overall, we believe that SIMPL2 has the potential to accelerate both PPI target mapping and drug discovery.

## Results

### Incorporation of tNLuc into the SIMPL system

Nanoluciferase (NanoLuc) is a small sized enzyme, originally discovered in deep sea shrimp, that is widely used in biochemical research (Hall et al, 2012; Oliayi et al, 2023). The activity of NanoLuc does not require ATP, and its engineered version uses furimazine as a substrate, producing the most brilliant luminescence among all the known luciferases. This makes it valuable as a highly sensitive reporter in many applications, including various PPI detection tools. The approach of splitting NanoLuc into fragments has also been used to study PPIs, exemplified by the two fragment NanoBiT approach (Dixon et al, 2016). Recent studies have further built upon this with the development of a new version of split NanoLuc, called tri-part NanoLuc (tNLuc) (Dixon et al, 2017; Ohmuro-Matsuyama and Ueda, 2018), where NanoLuc is split into three fragments: Δ11S and two C-terminal 11 amino acid peptides (β9 and β10). In this format, β9 and β10 are separately fused to bait and prey proteins. The luciferase is reconstituted upon bait/prey interaction in presence of Δ11S, allowing for production of luminescence upon addition of substrate. tNLuc is characterized by reduced background signal and minimized potential to perturb tagged proteins due to the small size of the β9 and β10 peptides (Oliayi et al, 2023). tNLuc has also been successfully applied in serological assays (Kim et al, 2022; Yao et al, 2021; Hall et al, 2021; Kim et al, 2021).

**Figure 1:**
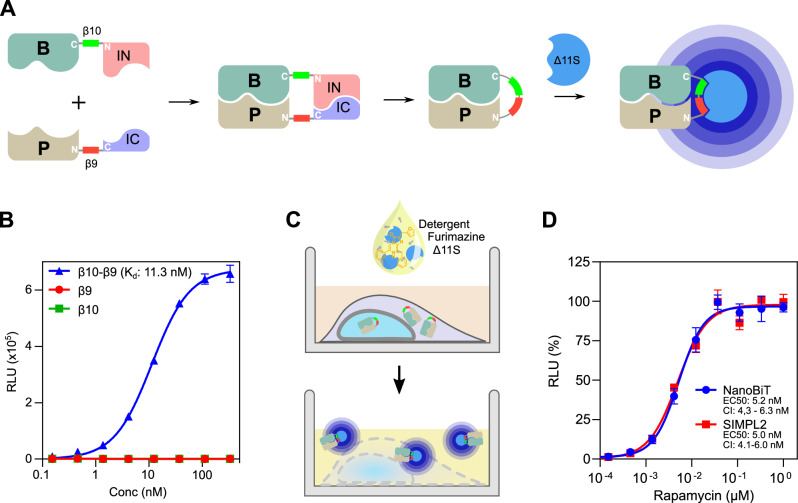
Overview and validation of SIMPL2 system. (**A**) In the SIMPL2 system, β10 and β9 tags derived from tNLuc are respectively included in the SIMPL constructs: immediately N-terminal to IN and immediately C-terminal to IC. Interaction-induced splicing conjugates the two tags and allows them to bind the third component of tNLuc, Δ11S. In the presence of the substrate furimazine, the reconstituted tNLuc produces luminescence signal. (**B**) Specificity of tNLuc. β9, β10 or β10-β9 peptide fused to recombinant protein (protein G) was serially diluted and incubated with Δ11S and furimazine, followed by luminescence measurements. Data are presented as mean ± s.d. with *n* = 4 biological replicates. The data were fit to a model of specific binding with Hill slope, with corresponding curves shown. (**C**) Schematic diagram of the cell-based SIMPL2 assay protocol. A liquid mixture of detergent, furimazine and Δ11S is added directly to the cultured cells expressing the SIMPL2 constructs being investigated, followed by incubation and luminescence detection. (**D**) Comparison of the SIMPL2 and NanoBiT assays performed using rapamycin-mediated FRB/FKBP1A interaction. Cells expressing FRB-β10-IN/FKBP1A-IC-β9 or FRB-LgBiT/FKBP1A-SmBiT were subject to rapamycin treatment with varied concentrations for two hours followed by measurement of reporter signal. Results are presented as mean ± SEM of four independent experiments with four technical replicates in each experiment. The data were fit to a four-parameter agonist-response model, with corresponding curves shown. Source data are available online for this figure.

NanoLuc executes its function in the homogeneous liquid phase without interference from unreacted/unbound components. This eliminates the need for the numerous cycles of aspiration and washing required by methods such as ELISA. We therefore decided to take advantage of this by incorporating tNLuc into the SIMPL system for PPI detection. For this, we inserted a β10 tag at the N-terminal side of the IN tag construct, and correspondingly inserted a β9 tag at the C-terminal side of IC tag construct (Fig. EV1A). It should be noted that a pair of common peptide tags for antibody recognition are still retained in the constructs for later validation of the system: a V5 tag in the IN construct and a FLAG tag in the IC construct. Interaction between the two tagged proteins of interest (‘B’ and ‘P’) induces splicing, ligating B and P together and arranging the β10 and β9 peptides in a tandem position. When Δ11S is externally added into the reaction system, it will bind the β10- β9 peptide to form a stable and active luciferase complex and produce luminescence signal. A schematic diagram of the new SIMPL2 system is provided in Fig. 1A. To validate our SIMPL2 system, we first characterized tNLuc in the SIMPL setting because the specificity and affinity of Δ11S had previously not been strictly assessed. Thus, the binding kinetics of Δ11S with different tNLuc tags were determined using recombinant protein G fused to β9 peptide alone, β10 peptide alone, or a tandem β10-β9 peptide (Fig. 1B). No significant binding was detected with β9 or β10 peptide tags alone within the measured concentration range (0.1–300 nM). In contrast, the β10–β9 peptide fusion tag resulted a binding curve with a K_d_ of 11.3 nM (Fig. 1B), suggesting strong affinity and exclusive specificity of Δ11S towards only β10–β9, but not β9 or β10 sequence. Therefore, the tNLuc readout should not be influenced by individually tagged protein partners in the absence of interaction.

**Figure EV1:**

SIMPL2 constructs. (**A**) A protein of interest “B” can be tagged with β10-IN on either its N- or C-terminus. Similarly, a second protein of interest “P” can be tagged with IC-β9 on either terminus. IN/IC handles are used for estimating protein expression. (**B**, **C**) Comparison of the SIMPL2 and SIMPL-ELISA assays performed using rapamycin-mediated FRB/FKBP1A interaction. Cells expressing FRB-β10-IN and IC-β9-FKBP1A (**B**) or FRB-β10-IN and FKBP1A-IC-β9 (**C**) were subject to rapamycin treatment with varied concentrations for 2 h followed by measurement of SIMPL2 signal. Data of SIMPL-ELISA with corresponding configuration combinations were extracted from previous study (*Nat Commun* (2020) 11:2440). The data were fit to a four-parameter agonist-response model, with corresponding curves shown. The raw data are normalized with corresponding maximal signals and presented as mean ± s.d. with *n* = 4 biological replicates for both SIMPL2 and SIMPL-ELISA in (**B**), *n* = 3 biological replicates for SIMPL2 and *n* = 4 biological replicates for SIMPL-ELISA in (**C**). Signal to background (S/B) ratios were calculated based on the derived models. Source data are available online for this figure.

Like our previous version of SIMPL, SIMPL2 involves co-expression of tagged ‘B’ and ‘P’ proteins of interest in cultured mammalian cells to allow the test PPIs to occur in their native environment. The subsequent detection of spliced protein is initiated by addition of a mixture of furimazine and Δ11S, alongside detergent which permeabilizes the cells to allow Δ11S uptake. After incubation, the luminescence signal is then recorded (Fig. 1C). We first evaluated the system using the rapamycin-induced dimerization of FKBP1A and FRB (the FKBP rapamycin-binding domain of mTOR) and compared it with the NanoBiT assay (Dixon et al, 2016). For this purpose, a stable SIMPL2 cell line of FRB-β10-IN/IC- β9-FKBP1A and a stable NanoBiT cell line of FRB-LgBiT/FKBP1A-SmBiT were created using CRISPR-Cas9. For the assay, the stable cells were simultaneously treated with rapamycin at different concentrations for two hours followed by measurement of either tNLuc or NanoBiT luminescence. Kinetics analysis revealed very similar EC50 values, 5.0 nM for SIMPL2 assay and 5.2 nM for NanoBiT assay, indicating their comparable performance in this rapamycin-induced interaction (Fig. 1D).

We also compared SIMPL2 with SIMPL coupled with ELISA in two formats: FRB-IN/IC-FKBP1A (Fig. EV1B) and FRB-IN/FKBP1A-IC (Fig. EV1C), and obtained EC50 values for rapamycin of 5.5 nM and 6.2 nM respectively in SIMPL2 assay. They are about one order of magnitude lower than those obtained using the SIMPL-ELISA platform (Yao et al, 2020), suggesting a significant improvement in the sensitivity. An approximately 5–6-fold improvement was also observed in the signal/background ratio for SIMPL2 versus the previous ELISA-based readout. We therefore conclude that coupling SIMPL with tNLuc substantially improves not only the operability of the assay but also its quantifiability.

### Evaluation of SIMPL2 using reference PPI sets

True SIMPL signal is potentially confounded by non-specific splicing derived from intrinsic affinity between the IN and IC intein fragments. To develop SIMPL, the GP41-1 split intein used was therefore engineered to minimize this intrinsic affinity to a residual level (Yao et al, 2020). However, if the proteins under study are sufficiently stable, even minimal levels of non-specifically spliced product could accumulate and generate background due to the irreversible nature of splicing. In this case, such non-specific splicing should be proportional to the abundance of the parental proteins. To test this hypothesis, we co-expressed β10-IN-tagged B proteins alongside an ‘IC handle’ construct consisting of only IC-β9, or similarly co-expressed IC-β9-tagged P protein alongside an ‘IN handle’ consisting of only β10-IN peptide (Fig. EV1A). The cell lysates were then subjected to Western analysis using anti-FLAG and anti-V5 antibodies to identify total vs. spliced protein products (Fig. EV2A,B). Here, the splicing signals reflect only the basal interactions driven by IN/IC affinity because the IN and IC handles do not contain any extra protein sequences. As expected, the blots showed that splicing signals were well correlated with total protein B or P expression. This observation suggests it is necessary to distinguish true PPI signal from background signal resulting from varied expression of the involved proteins. On the other hand, the signal produced by IN/IC handles could also be used as to estimate protein expression, which can be harnessed to help modify SIMPL2 signal to provide a better measurement of true interactions.

**Figure EV2:**

**Figure 2:**
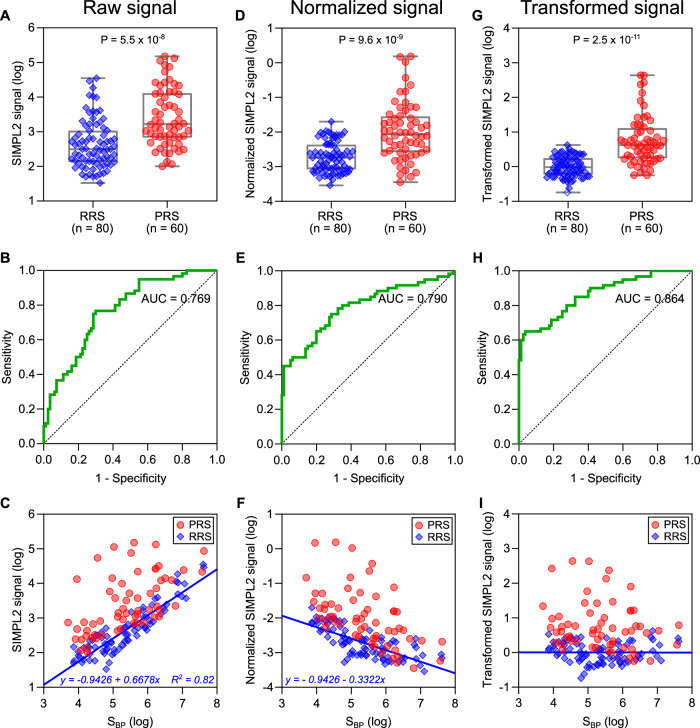
Reference set benchmarking of SIMPL2 as a PPI discovery tool. SIMPL2 DNA constructs in B-β10-IN and IC-β9-P format encoding each protein pair from the RRS (*n* = 80) and PRS (*n* = 60) were co-transfected into HEK 293T cells. Interaction between B and P proteins was measured as luminescence signal. In parallel, each B-β10-IN construct was co-expressed with IC handle and the obtained luminescence signal (S_B_) was used as an estimate of protein B expression. Similarly, protein P expression (S_P_) was estimated by IC-β9-P and IN handle co-expression. Experiments were performed in triplicates and their average is presented as a single point. (**A**, **B**) Comparison of RRS and PRS results using raw data (in the logarithmic scale) was performed via box-whisker plot (**A**) and ROC analysis (**B**). AUC area under curve. (**C**) Comparison of interaction signals (logarithmic scale) and expression signals (S_BP_, product of B and P handle signals in logarithmic scale) is presented as a scatter plot. Simple linear regression analysis of RRS data was performed and the resulting fitted function is presented inset. The interaction signals were then normalized with B and P expression values using the formula *S*_*normalized*_*= S*_*raw*_*- S*_*BP*_ in which S_raw_ is the raw SIMPL2 signal and all terms are in logarithmic scale, or equivalently calculated as *s*_*normalized*_*= s*_*raw*_*/ (s*_*b*_*x s*_*p*_*)* in which all terms are in linear scale. Analyses of normalized data are presented in box-whisker plot (**D**), ROC plot (**E**) and scatter plot (**F**). Alternatively, interaction signals were also transformed according to the empirical function derived from the linear distribution of RRS signals in (**C**), using the formula *S*_*transformed*_*= S*_*raw*_ + *0.9426 – 0.6678S*_*BP*_. Analyses of transformed data are presented in a box-whisker plot (**G**), ROC plot (**H**) scatter plot (**I**). In (**A**, **D**, **G**), boxplot center lines are medians, box limits are 25th and 75th percentiles, minima are smallest data points, and maxima are largest data points. In RRS, *n* = 80. In PRS, *n* = 60. *P* values obtained from two-tailed student’s t test were presented. Source data are available online for this figure.

Behavior of reference set in the SIMPL2 system. (**A**) β10-IN-tagged Protein ‘B’ constructs were co-expressed with IC handle in HEK 293T cells and the cell lysates were subjected to Western blot analysis. The total expression level of B was detected using α-V5 antibody, while levels of spliced product caused by non-specific interaction with IC handle were detected using α-FLAG antibody. (**B**) IC-β9-tagged Protein ‘P’ constructs were co-expressed with IN handle, followed by western blot analysis using α-V5 antibody and α-FLAG antibody, to detect protein expression levels as described in (**A**). (**C**, **D**) Interaction signals of RRS and PRS in Fig. 2 were plotted against ‘handle’ signal correlated with B protein expression (S_B_ in logarithmic scale) (**C**) or P protein expression (S_P_ in logarithmic scale) (**D**). (**E**, **F**) Evaluation of different data processing methods. Based on the ROC curves obtained in Fig. 2B, E, H, sensitivities of different data processing methods at selected specificities (1.00, 0.98, 0.95 or 0.90) are shown in (**E**). Similarly, specificities of the methods at selected sensitivities (0.2, 0.4, 0.4 or 0.8) are shown in (**F**). (**G**) Comparison of SIMPL2 and SIMPL-ELISA. ROC curve of a SIMPL-ELISA assay with a PRS set (*n* = 88) and an RRS set (*n* = 88) (published in *Nat Commun* 11:2440) is compared with that from a SIMPL2 assay using the same reference sets. Source data are available online for this figure.

We next used reference PPIs to evaluate the performance of the SIMPL2 system. A *Homo sapiens* positive reference set version 1 (hsPRS-v1) was previously created for the purpose of method evaluation (Venkatesan et al, 2009). The hsPRS-v2 (*n* = 60) is an upgraded version with removal of interaction pairs with false annotations or of incorrect DNA sequences (Choi et al, 2019). Since it is well accepted (Trepte et al, 2024), hsPRS-v2 was used as the PRS in our current study. The corresponding random reference set (RRS) contains 80 protein pairs with baits and preys selected from hsPRS-v1 but used in combinations computationally determined to have a low probability of interaction. In the same experiment, each bait or prey was also tested with corresponding IN/IC handle to obtain an estimate of its expression. Comparing the SIMPL2 results using the PRS vs. RRS revealed a statistically significant difference in the overall raw mean signal between the two datasets (Fig. 2A). Receiver operating characteristic (ROC) analysis (Fig. 2B) produced an area under curve (AUC) value of 0.769. However, overlap in signal between the two reference sets was high (Fig. 2A), making it challenging to distinguish more true positives from background.

We next used scatter plots to compare the SIMPL2 signals obtained with the reference sets to the corresponding expression of proteins B (S_B_, Fig. EV2C) or P (S_P_, Fig. EV2D) estimated by co-expressing B and P proteins with IC or IN handle, respectively. Notably, PRS vs. RRS did show certain separation in this two-dimensional space. This separation was further improved upon plotting against the product of B and P expressions (S_BP_, or S_B_ + S_P_ since they are in logarithmic scale in the plot, Fig. 2C), suggesting that normalizing SIMPL2 signal based on B and P expression could improve assay performance. Thus, we normalized the SIMPL2 signals by dividing them by the product of the B and P expression values. This normalization made the RRS signals more convergent, allowing better separation of the two sets as shown in a box-whisker plot (Fig. 2D). ROC analysis also demonstrated a mild improvement in AUC (AUC = 0.790) (Fig. 2E).

We noted that, in the scatter plot of SIMPL2 vs. S_BP_ (Fig. 2C), RRS values were distributed in a tight linear pattern (*R*^2^ = 0.821). This fit confirms a strong relationship between estimated protein expression level and background signal, and more importantly the linear distribution supports the assumption that all RRS pairs follow the same non-specific kinetics. However, the fitted linear regression equation of raw RRS signals presented a slope far different from 1.0 (Fig. 2C), and correspondingly normalization using S_BP_ did not bring RRS to an even level (Fig. 2F), suggesting the process deviates from the standard second-order mass action kinetics, a model laying the basis for the normalization approach. We reasoned that the empirical regression equation of RRS (Fig. 2C) may more precisely describe the true behavior of non-specific binding, and therefore it can be used to remove the contribution of non-specific binding to SIMPL2 signal. Indeed, performing an RRS-based transformation flattened the RRS signal fit in the scatter plot (Fig. 2I) and produced maximal convergence in RRS signal values, thereby allowing for the best separation (Fig. 2G). ROC analysis furthermore demonstrated a significantly improved AUC (0.864) (Fig. 2H). The performance difference among these three data processing methods can be further intuitively seen by comparing the sensitivity values obtained at certain specificity (Fig. EV2E), or the specificity values obtained at certain sensitivity (Fig. EV2F), derived from the ROC curves (Fig. 2B,E,H): the transformed set showing the best sensitivity and specificity in all conditions.

Finally, we compared the performance of SMIPL2 with SIMPL-ELISA. Our pervious SIMPL-ELISA analysis used a PRS set containing 88 protein pairs and an RRS set of 88 pairs (Yao et al, 2020). A SIMPL2 assay on the same reference sets presented a ROC curve of transformed data with slightly better AUC than SIMPL-ELISA (0.86 for SIMPL2 v.s. 0.81 for SIMPL-ELISA) (Fig. EV2G). However, a significant improvement was observed in sensitivity when maintaining a high specificity. For example, at the point of 100% specificity, SIMPL2 allowed a ~50% sensitivity while only ~33% was achieved in SIMPL-ELISA assay. Thus, this framework of RRS-based transformation appears to be an effective approach for enhancing the sensitivity and specificity of SIMPL2 assay.

### SIMPL2 as an efficient tool for identifying PPI modulators

The operational, quantitative, and modular features of SIMPL2 suggest that it can potentially work as a powerful cell-based platform for identifying PPI modulators. Since stable cells are best suited for this type of application (as their use both simplifies assay setup and reduces variability) we developed cell lines stably expressing target protein pairs of interest in SIMPL2 format. To prevent the occurrence of target PPIs before treatment with PPI modulators, expression of the SIMPL2 constructs was placed under the control of a Tet-on promoter, which can only be induced by addition of doxycycline.

**Figure 3:**
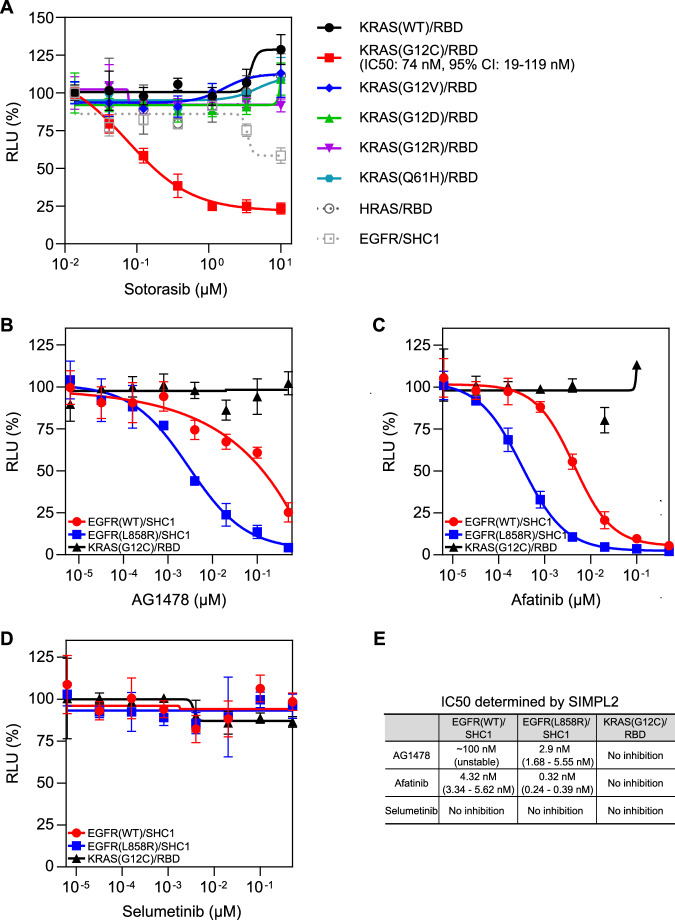
Evaluation of SIMPL2 as a drug discovery tool for PPI inhibitors. (**A**) Characterization using sotorasib, a KRAS G12C-specific inhibitor. HEK 293T cells stably expressing IC-β9-KRAS (HEK 293 WT, G12C, G12D, G12R, G12V or Q61H mutant) or IC-β9-HRAS and β10-IN-RBD, or cells expressing EGFR-IN-β10 and IC-β9-SHC1 were treated with doxycycline and sotorasib at the indicated concentrations for 16 h, followed by SIMPL2 measurement of luciferase signal. SIMPL2 readouts were then normalized as described in “Methods”. Data are presented as mean ± s.d. with *n* = 4 biological replicates. The data were fitted into a four-parameter inhibitor-response model. (**B**–**E**) Characterization using EGFR inhibitors. HEK 293 cells stably expressing EGFR(WT)-β10-IN/IC-β9-SHC1 or EGFR(L858R)-β10-IN/IC-β9-SHC1, or control cells expressing IC-β9-KRAS(G12C)/RBD-β10-IN were treated with the EGFR inhibitors AG1478 (**B**) or afatinib (**C**), or the MEK inhibitor selumetinib (treatment control) (**D**) at the indicated concentrations together with doxycycline (0.5 μg/ml) for 16 h, followed by SIMPL2 measurement of luciferase signal. SIMPL2 readouts were then normalized as described in Methods. Data are presented as mean ± s.d. with *n* = 4 biological replicates. The data were then fitted into a four-parameter inhibitor-response model, with calculated IC_50_ values for each inhibitor/PPI pair presented in (**E**). The 95% CI of each IC_50_ is also included in parenthesis. Source data are available online for this figure.

**Figure EV3:**
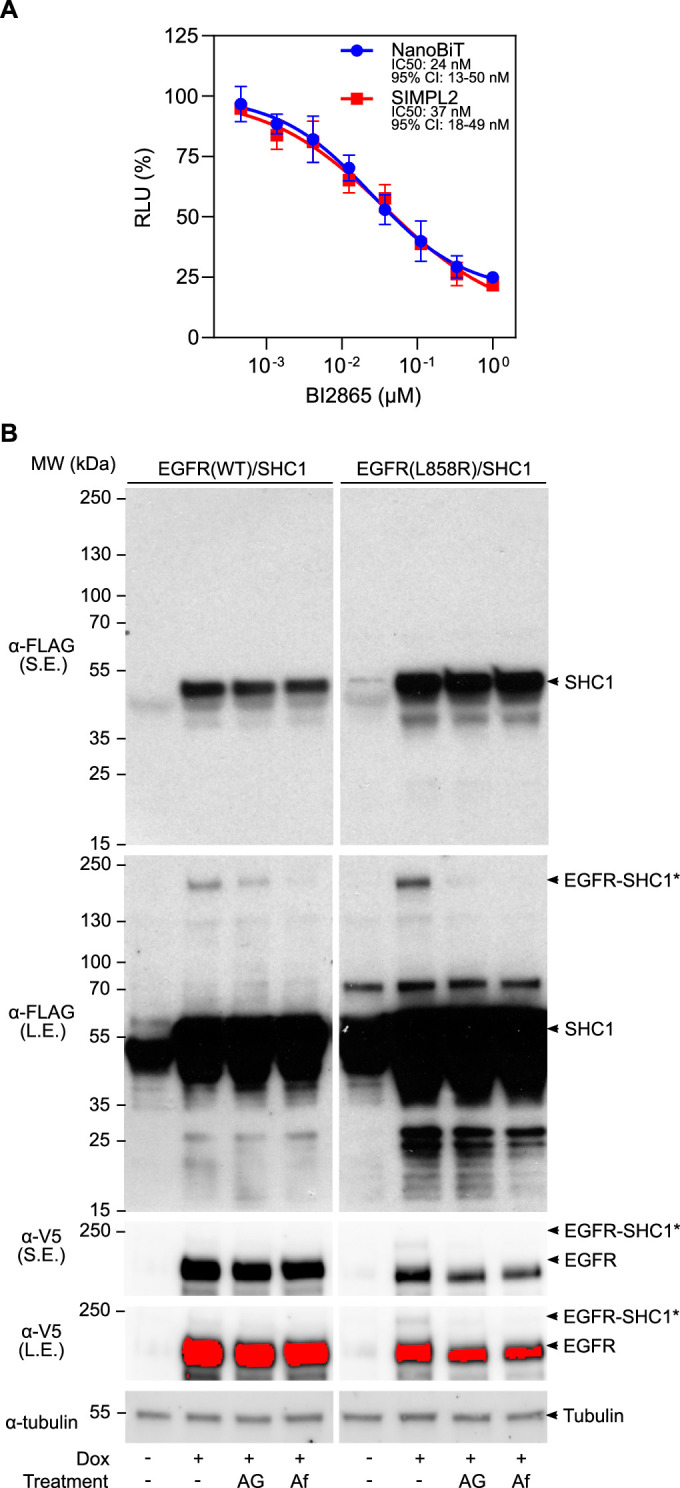
Validation of SIMPL2 as method for detecting PPI inhibition. (**A**) Comparison of SIMPL2 and NanoBiT in evaluating the KRAS inhibitor BI2865. SIMPL2 stable cells expressing IC-β9-KRAS(WT)/β10-IN-RBD were simultaneously treated with both doxycycline and BI2865 for 5 h at the indicated concentrations followed by luminescence measurement. In contrast, the expression of SmBiT-KRAS(WT)/LgBiT-RBD was induced for 16 h in NanoBiT stable cells, followed by BI2865 treatment. Data are presented as mean ± s.d. with *n* = 4 biological replicates and were fitted into a four-parameter inhibitor-response model. (**B**) The expression of EGFR (WT or L858R)-β10-IN and IC-β9-SHC1 in stable cells was induced with doxycycline plus treatment with AG1478 (AG, 100 nM) or afatinib (Af, 100 nM). After 16 h of treatment, the cells were lysed and subjected to western blot analysis. Both blots with short exposure (S.E.) and long exposure (L.E.) are presented. Interaction-induced splicing produced a fusion protein of EGFR-SHC1 (*). Source data are available online for this figure.

We first evaluated the performance of SIMPL2 using the mutant specific KRAS G12C inhibitor sotorasib (AMG 510), which irreversibly inhibits KRAS G12C mutant through covalent binding, locking it into an inactive conformation and thereby preventing interaction with RAF proteins (Canon et al, 2019). For our study, we created several reporter cell lines stably expressing IC-β9-KRAS (WT or oncogenic KRAS mutants: G12C, G12D, G12R, G12V and Q61H) and the Ras-binding domain (RBD) of RAF1 in the β10-IN-RBD format. Nonrelated cell lines expressing HRAS/RBD or EGFR/SHC1 were used as controls. Expression of the target proteins was induced by doxycycline in the presence of co-treatment with different concentrations of sotorasib and the target interactions were evaluated as SIMPL2 luciferase signals (Fig. 3A). The efficacy of sotorasib on KRAS G12C mutant was verified as a dose-responsive inhibition of KRAS/RBD interaction signal with an IC50 of 74 nM, a value consistent with previous reports (Canon et al, 2019). In contrast, no apparent inhibition of WT KRAS or other mutants was observed within the range of sotorasib concentrations tested, consistent with known sotorasib specificity (Canon et al, 2019). Importantly, no significant inhibition was observed in HRAS/RBD and EGFR/SHC1 cells. SIMPL2 was further compared with NanoBiT using the pan-KRAS inhibitor BI2865 (Kim et al, 2023) (Fig. EV3A). The high similarity of the obtained IC50 values (37 nM for SIMPL2 and 24 nM for NanoBiT) demonstrates that the assays have comparable performance in evaluating RAS inhibitors.

We also evaluated two EGFR tyrosine kinase inhibitors (TKIs), AG1478 (tyrphostin), a prototype EGFR inhibitor that functions through competitive binding to ATP-binding sites (Yaish et al, 1988), and afatinib, a second-generation EGFR inhibitor operating through irreversible binding to EGFR (Li et al, 2008). These molecules indirectly inhibit EGFR PPIs by antagonizing EGFR tyrosine kinase activity, blocking its autophosphorylation and subsequent interaction with downstream signaling molecules such as SHC1. Two reporter cell lines stably expressing EGFR (WT or oncogenic L858R mutant)-β10-IN and IC-β9-SHC1 were created for this study to examine whether these inhibitors exert differential effects on WT and the oncogenic mutant. The EGFR/SHC1 interaction was evaluated as SIMPL2 luciferase signal while treated with different concentrations of the EGFR inhibitors, revealing a distinct dose-responsive inhibition of EGFR/SHCI interaction signal (Fig. 3B,C). More specifically, AG1478 inhibited WT EGFR with an IC_50_ of ~100 nM, and L858R mutant with 2.9 nM, while afatinib displayed more potent inhibition, with an IC_50_ of 4.32 nM for WT EGFR and 0.32 nM for L858R mutant (Fig. 3E) which are in line with previously reported profiles (Hirano et al, 2015). As expected, neither compound showed any significant inhibitory effect on the KRAS (G12C)/RBD interaction within the tested concentration range (Fig. 3B,C,E). Treatment of the same set of cells with selumetinib (AZD6244), a MEK inhibitor, was performed here as a negative control, and showed no effect on the interaction of either EGFR WT or L858R mutant (Fig. 3D). The inhibitory effects of AG1478 and afatinib on WT and L858R EGFR were also verified by western blot analysis, in which the splicing between EGFR and SHC1 was reduced or abolished by inhibitor treatment in a trend consistent with the SIMPL2 results (Fig. EV3B). Collectively, these results demonstrate the ability of SIMPL2 to detect the specific action of inhibitors against their corresponding targets, making it a potentially useful tool for rapid PPI- and enzymatic-inhibitor discovery.

**Figure 4:**
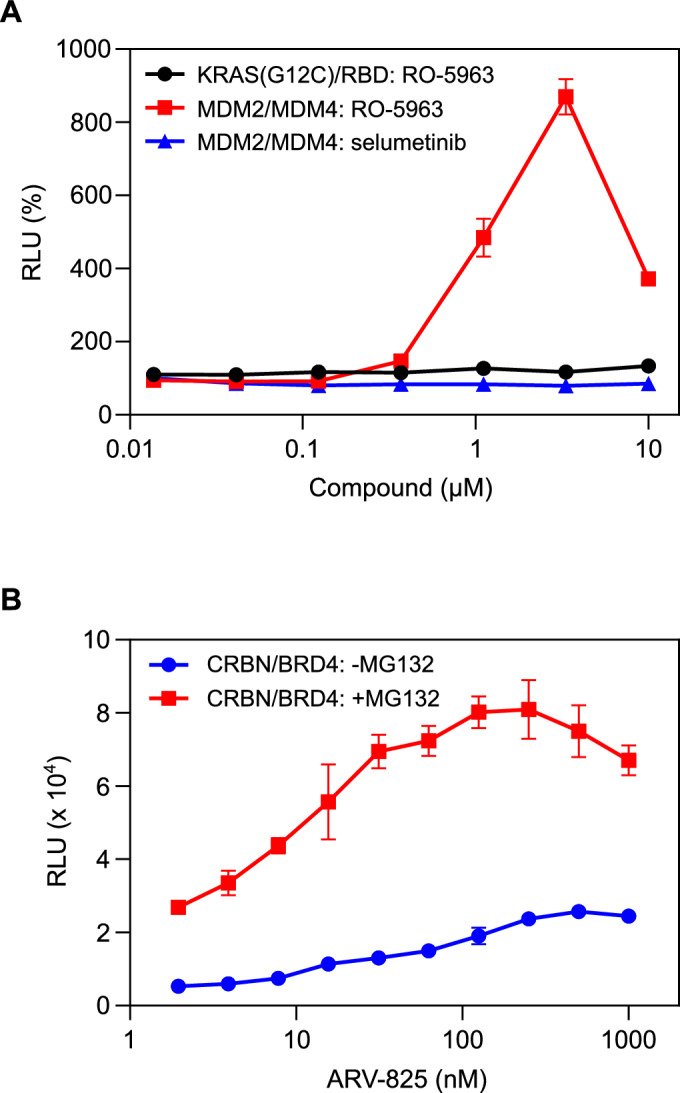
Evaluation of SIMPL2 as a drug discovery tool using molecular glue and PROTACs. (**A**) HEK 293T cells stably expressing MDM2-β10-IN/IC-β9-MDM4 or control cells expressing IC-β9-KRAS(G12C)/RBD-β10-IN were treated with RO-5963 or selumetinib (compound control) at the indicated concentrations together with doxycycline (0.5 μg/ml) for 16 h, followed by SIMPL2 measurement of luciferase signal. Samples were measured and normalized as described in Materials and Methods. Data are presented as mean ± s.d. with *n* = 4. (**B**) β10-IN-CRBN/IC-β9-BRD4 expression in the stable HEK 293 cells was induced with doxycycline (0.5 μg/ml) for 16 h, followed by 3 h treatment of ARV-825 at different concentrations, in the presence and absence of MG132 proteasome inhibitor (1 μM). The cells were then subjected to SIMPL2 luciferase measurement, and the raw values are plotted. Data are presented as mean ± s.d. with *n* = 3. Source data are available online for this figure.

We next tested the system with molecular glues, which are small molecule compounds that induce target protein interaction. In our current study, we used rapamycin to characterize SIMPL2 (Fig. 1D). Notably, the natural product rapamycin is a well-studied molecular glue for FKBP12A/FRB dimerization, and our results support the suitability of SIMPL2 for measuring the activity of molecular glues. However, we also tested another molecular glue, RO-5963 (Graves et al, 2012), which operates through a mode of action distinct from that of rapamycin. As a p53 inhibitor, RO-5963 induces homo- or hetero-dimerization between MDM2 or MDM4 (MDMX). For our experiments, cells co-expressing MDM2-β10-IN and IC-β9-MDM4 were treated with RO-5963 at different concentrations and then subjected to the SIMPL2 assay (Fig. 4A). The MDM2/MDM4 interaction was observed at RO-5963 concentrations above 0.3 μM, reaching a peak at 3 μM (Fig. 4A, red line). The interaction was not observed, however, when cells were treated with control compound selumetinib (Fig. 4A, blue line). Control interaction between KRAS G12C and RBD was also not affected upon RO-5963 treatment, demonstrating the ability of SIMPL2 to detect the specificity of RO-5963 (Fig. 4A, black line). Interestingly, the interaction started to decline above 3 μM. This hook effect, however, can be explained by the mode of action of RO-5963 (Graves et al, 2012). To clarify, the two protein molecules are anchored together through a pair of dimerized RO-5963 molecules. When excess RO-5963 molecules are present, however, the balance of binding reaction is driven toward the production of mono-protein bound RO-5963 molecules. In contrast, rapamycin engages its targets most likely through a sequential pathway, wherein it first binds to FKBP12A followed by the interaction with FRB (Banaszynski et al, 2005). Therefore, its binding kinetics display a monophasic change.

**Figure EV4:**
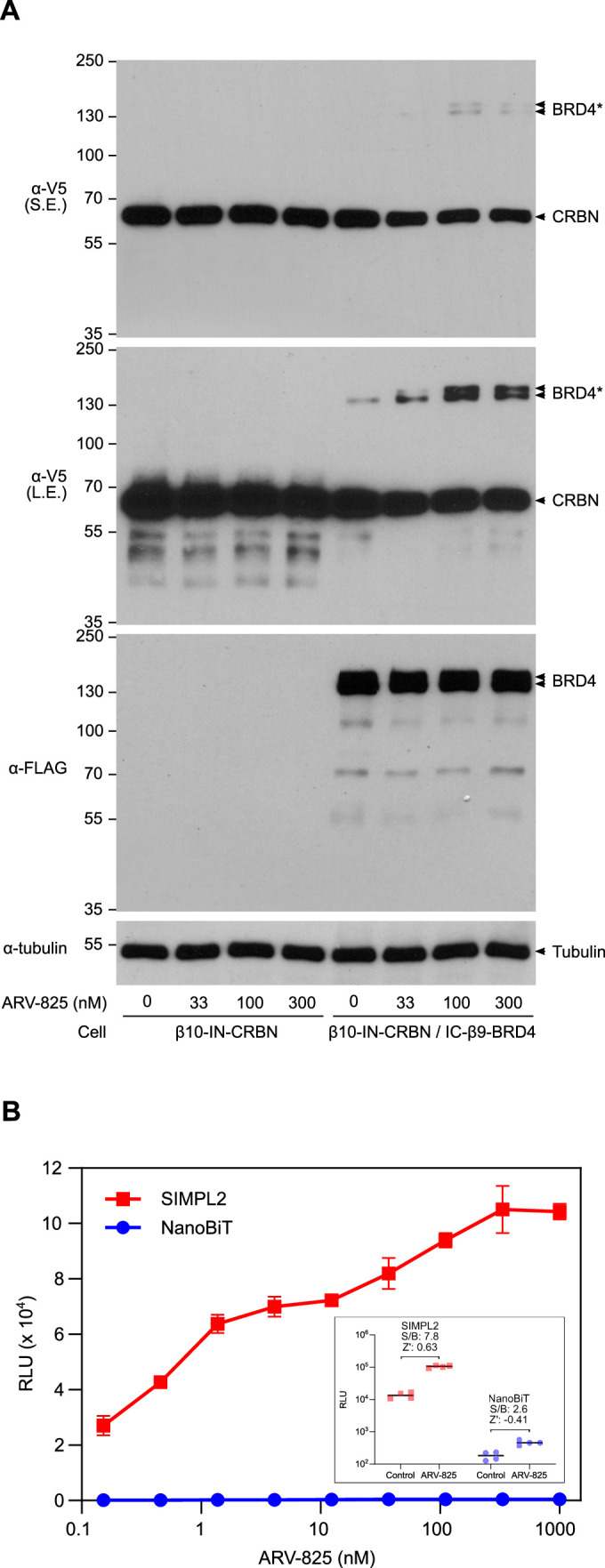
Validation of SIMPL2 as method for detecting PROTACs using a Western blot readout. (**A**) The expression of β10-IN-CRBN/IC-β9-BRD4 in stable cells was induced with doxycycline for 16 h followed by treatment with ARV-825 at the indicated concentrations for 3 h. The cells were lysed and subjected to Western blot analysis. Stable cells expressing only β10-IN-CRBN were used as a control. ARV-825 induced splicing allowed V5 tag transfer to BRD4, which presents a BRD4 band (*) in the V5 blot. (**B**) Comparison of SIMPL2 and NanoBiT in evaluating PROTAC. Stable cells expressing β10-IN-CRBN/IC-β9-BRD4 or SmBiT-CRBN/LgBiT-BRD4 were treated with ARV-825 at the indicated concentration for three hours followed by signal measurement. Data are presented as mean ± s.d. with *n* = 4 biological replicates. S/B signal/background ratio. Source data are available online for this figure.

We also examined the ability of SIMPL2 to monitor the activity of PROTACs. These molecules are different from molecular glue, consisting of two active heads (one for binding target and the other for binding E3 ligase) linked through a linear chain, and are specialized for use in directing targets for ubiquitination and degradation by the proteasome. We evaluated SIMPL2 performance for characterizing PROTACs using ARV-825, a well characterized PROTAC that recruits the bromodomain protein BRD4 to the E3 ligase cereblon (CRBN) (Lu et al, 2015). Cells co-expressing β10-IN-CRBN and IC-β9-BRD4 were treated with ARV-825 at different concentrations (Fig. 4B). Since ARV-825 treatment leads to proteasome-mediated BRD4 degradation, a set of cell samples were also co-treated with the proteasome inhibitor MG132. A positive correlation between ARV-825 concentration and BRD4/CRBN interaction was observed, verifying the activity of ARV-825 (Fig. 4B). The same effect was also verified using Western blot analysis (Fig. EV4). The SIMPL2 signals were profoundly enhanced by MG132 treatment, further confirming the in vivo functionality of ARV-825. On the other hand, PROTACs usually produce hook effects at high concentration (Pettersson and Crews, 2019), which was also captured by the assay especially when MG132 was added. We further compared the capability of NanoBiT in detecting the activity of ARV-825 (Fig. EV4B). Although ARV-825-induced proximity could be captured by NanoBiT, the signals were low (two orders of magnitude lower than SIMPL2 signals), leading to a low signal/background ratio, 2.6 v.s. 7.8 produced by SIMPL2. This result suggests a potential difficulty of NanoBiT in producing statistically significant data in high throughput studies involving weak PPIs or induced proximity, however further work with additional candidates will need to be performed in order to determine the extent of this difference.

Based on the above results, we therefore conclude that SIMPL2 is well-suited for the sensitive detection and monitoring of both PROTACs and molecular glues, as well as PPI inhibitors.

## Discussion

In our current study, we report the development of the SIMPL2 platform, coupling our original SIMPL system to an improved tNLuc readout. This new platform offers advantages over our earlier approach. For example, in the new system reactions occur in the homogenous liquid phase, making the process quick and easy to perform. Additionally, expensive reagents such as labelled antibodies are not required, greatly reducing cost. This makes the SIMPL2 system more labor- and cost-effective (and better suited for use in HTS formats) than the original SIMPL, which relied on Western blotting, ELISA or HTRF readouts.

One challenge with SIMPL is the non-specific signals caused by low intrinsic affinity between the IN and IC intein fragments used in the tags. Although the affinity has been greatly reduced via targeted protein engineering, it is still possible for fusion product to accumulate to non-negligible levels when using very highly expressed proteins, due to the irreversible nature of intein-mediated splicing. Taking the advantage of the strong quantifiability provided by the SIMPL2 system, however, we were able to set up a framework using this background as an indirect measure of protein expression, which could be used to modify signal and help distinguish true vs non-specific PPIs. This framework was evaluated using benchmark PPI reference sets and provided results favouring signal from the PRS vs. the RRS; specifically, 48% of PPIs within the PRS were identified at a threshold where no RRS interactions were reported (with ROC analysis presenting an AUC value of 0.86). These parameters demonstrate good behavior of SIMPL2 in detecting true PPIs, although they cannot be directly compared to those obtained from other methods (Choi et al, 2019; Braun et al, 2009; Trepte et al, 2018) due to the differences in the RRS employed. It should also be noted that there are a total of eight different combinations of bait/prey tagging orientation possible: B-β10-IN/IC-β9-P, B-β10-IN/P-IC-β9, β10-IN-B/IC-β9-P, β10-IN-B/P-IC-β9, IC-β9-B/P-β9-IN, IC-β9-B/β9-IN-P, B-IC-β9/P-β9-IN and B-IC-β9/β9-IN-P. In this proof-of-technology study, however, we only explored one tagging strategy. Thus, the sensitivity can potentially be further improved in future work via exploration of all possible tagging orientations as demonstrated by previous studies (Trepte et al, 2018; Choi et al, 2019). Thus, we conclude that SIMPL2 can be readily applied in high throughput PPI discovery by screening IN- or IC-fused open reading frame (ORF) libraries in an array-based format.

We also validated our SIMPL2 system for use as drug discovery tool. With the advances in our understanding of PPI mechanisms, and the importance of these mechanisms in mediating disease states, it is now more important than ever to translate this knowledge into practical medical applications. To this end, new strategies for manipulating PPIs have opened up avenues for hitting disease targets previously considered undruggable. Indeed, PPI targeted drug discovery has recently become an intensive area of research in both the pharmaceutical industry and academia, and PPI detection methods have become increasingly important players in the early phases of this type of drug discovery. However, although numerous PPI methods are available, many of them are not ideally suited for hit identification and characterization. In particular, traditionally employed biophysical and biochemical techniques are accompanied by certain limitations such as high cost, requirement for labeling, inability to recapitulate in vivo conditions, incompatibility with HTS formats and the requirement for highly specialized equipment and expertise, all of which have the potential to slow the pace of drug discovery.

In contrast, new drug discovery systems such as SIMPL2 provide promising alternatives to help improve the drug discovery paradigm. For instance, the incorporation of the tNLuc readout in SIMPL2 bestows an improved operability and quantifiability, which enhances the scalability of the system to an HTS test format. We have also shown how the system is capable of monitoring different types of PPI modulators, including inhibitors, molecular glues and PROTACs, all within a live cell format that provides immediate feedback on important parameters like toxicity and permeability. Furthermore, the modular design of SIMPL2 allows it to be rapidly adapted to different types of protein targets, irrespective of their cellular localization.

The last few years have also seen considerable development and maturation of artificial intelligence (AI) and quantum computing (Vakili et al, 2024), and its active participation in drug development (Arnold, 2023; Marissa Mock et al, 2023). The power of AI and quantum computing has the potential to greatly alter drug discovery in the future. However, candidates developed by AI/quantum computing-based drug discovery approaches still need a means of rapid experimental validation which avoids a possible bottleneck step in the discovery process. A highly scalable, labor- and cost-effective system such as SIMPL2 may be suitable for such a role.

It should be noted that SIMPL2 does not come without limitations, however. There are several important issues that need to be considered especially for PPI modulator studies. Firstly, unlike some assays like NanoBiT which can follow PPI kinetics in a real-time manner, SIMPL can only capture interaction ‘snapshots’. Secondly, the SIMPL reporter mechanism involves an irreversible process which captures only protein association but not dissociation. Once splicing occurs, it can not be broken up by an inhibitor. Thus, it is essential to treat cells with an inhibitor ahead of target protein expression. Thirdly, various PPIs may behave differently in SIMPL. Those with fast association kinetics may rapidly undergo splicing in SIMPL and thereby be unsuitable for inhibitor discovery. Finally, like any cell-based assay, SIMPL can sometimes be confounded by off-target effects on cellular activities such as cell viability, transcription, translation, luciferase activity *etc*. As such, it is imperative that all experiments be well-designed, employing proper controls and quick orthogonal validation of top candidates, to help distinguish direct and indirect modes of action.

Overall, however, we believe that our new SIMPL2 system offers a wide range of benefits that should make it a suitable tool for advancing both PPI studies and drug discovery endeavors.

## Methods

**Table:** Reagents and tools table

Reagent/resource	Reference or source	Identifier or catalog number
**Experimental models**		
HEK 293T (*Homo sapiens*) cells	ATCC	CRL-3216
**Recombinant DNA**		
pZY1001	This study	N/A
pZY1003	This study	N/A
pZY1004	This study	N/A
pZY1006	This study	N/A
pZY1007	This study	N/A
pZY1008	This study	N/A
pZY1009	This study	N/A
pZY1010	This study	N/A
pDONR223	ThermoFisher	
pX330	Addgene	158973
**Antibodies**		
Anti-FLAG	Sigma-Aldrich	F1804
Anti-V5	Cell Signaling Technology	13202
Goat anti-mouse HRP	Cell Signaling Technology	7076
Goat anti-rabbit HRP	Cell Signaling Technology	7074
**Oligonucleotides and other sequence-based reagents**		
ZY1462	This study	N/A
ZY2447	This study	N/A
U6	Eurofins	N/A
**Chemicals, enzymes and other reagents**		
Gateway Gene LR Clonase II Enzyme mix	ThermoFisher	11791100
Gateway Gene BP Clonase Enzyme mix	ThermoFisher	11789021
CloneAmp HiFi PCR premix	Takara	639298
Gene Assembly Master mix	New England BioLabs	E2611
Linear polyethylenimine (PEI MAX), 40 kDa	Polysciences	24765‐2
DMEM high glucose	ThermoFisher	41965062
OptiMEM	ThermoFisher	11058021
384 well cell culture plate	Greiner Bio-One	781080
Nickel Sepharose	Cytiva	17526801
Sotorasib	Selleckchem	S8830
BI2865	MedChemExpress	HY-153724
Afatinib	Selleckchem	S1011
AG1478	Cell Signaling Technology	9842
Selumetinib	Selleckchem	S1008
RO-5963	MedChemExpress	HY-120086
ARV-825	Cayman Chemical	1818885-28-7
MG132	Selleck	S2619
Rapamycin	Selleckchem	S1039
Nickel Sepharose	Cytiva	17526801
**Software**		
GraphPad Prism 10	https://www.graphpad.com	
**Other**		
Synergy LX plate reader	Biotek	
Clariostar plate reader	BMG Labtech	
MultiFlo FX Multimode Dispenser	BioTek	
ChemiDoc MP Imaging System	Bio-Rad	

### Molecular cloning and library preparation

Four SIMPL2 vectors with different orientations of GP41-1 split intein and tNLuc tags were used in this study (Appendix Fig. S1A). All of them contain an attR cassette compatible for LR Gateway cloning. pZY1001: attR-β10-IN. pZY1004: β10-IN-attR. pZY1003: IC-β9-attR. pZY1006: attR-IC-β9. These cassettes are under the control of the third generation Tet-On promotor. In addition, all the vectors contain an expression cassette including RPBSA promoter, rtTA, P2A and an antibiotics marker, which is either HygR in pZY1001 and pZY1004, or PuroR in pZY1003 and pZY1006. Similarly, NanoBiT vectors were created: pZY1007: LgBiT-attR; pZY1008: attR-LgBiT; pZY1009: SmBiT-attR; pZY1010: attR-SmBiT (Appendix Fig. S1A). The above vectors were generated using Gibson assembly. Most cDNAs of target genes were originally obtained from human ORFeome collection or from the Openfreezer collection at Lunenfeld-Tanenbaum Research Institute. Those not in entry clone vectors were inserted into pDONR223 by PCR and BP cloning. Different cDNAs were then cloned into vectors by LR reactions. Some DNA constructs are listed in Appendix Fig. S1B. All cDNA sequences were verified using Sanger sequencing. CRISPR plasmids were prepared by insert guide sequence into pX330 vector as described (Ran et al, 2013). Guide sequences used in this study are listed as follow:

CCR5: TAATAATTGATGTCATAGAT

ROSA26: GTCGAGTCGCTTCTCGATTA

Temp2 (specific cleavage site in SIMPL2 vector): GATTTCTTCTTGCGCTATCT.

### Cell culture and treatment

HEK 293T cells were grown in DMEM supplemented with 10% fetal calf serum. For SIMPL2 assay, cells were seeded in 384-well plates with 5000–10,000 cells per well. For western blot analysis, HEK 293T cells were seeded in 24-well plates and transfected with various plasmids with polyethylenimine (PEI). After 24 h of transfection, the cells were treated with doxycycline (1 µg/ml) for overnight followed by lysis and western blot analysis. The cells used in this study were not recently authenticated or tested for mycoplasma contamination.

### Stable cell preparation

HEK 293T cells were grown in a 24-well plate. Mix a SIMPL2 plasmid (125 ng), Temp2-pX330 (62.5 ng) and CCR5/ROSA26-pX330 (62.5 ng) with 0.75 µl PEI in 0.25 ml PBS and incubate for 30 min. The DNA/PEI mixture was added in the cell culture. After 24 h, the cells were trypsinized and reseeded in a 10 cm plate with a medium containing hygromycin or puromycin according to the antibiotics-resistant marker in the SIMPL2 plasmid. After colonies appeared, they were individually picked followed by validation of protein expression.

### Western blot analysis and immunoprecipitation

Cells were lysed in buffer H (Triton X 100 1%, β-glycerophosphate pH 7.3 50 mM, EGTA 1.5 mM, EDTA 1 mM, orthovanadate 0.1 mM, DTT 1 mM supplemented with protease inhibitors (Roche)). After centrifugation at 21,000 × *g* for 10 min, the supernatants were mixed with Laemmli sample buffer, boiled at 95 °C for 3–5 min and subjected to Western blot analysis. Antibodies used for Western blot analysis were: α-FLAG antibody purchased from Sigma-Aldrich (F1804) with 1:10,000 dilution and α-V5 antibody from Cell Signaling Technology (#13202) with 1:10,000 dilution. Images were acquired using ChemiDoc MP system and its accompanied software Image Lab Touch.

### Specificity of Δ11S

Recombinant proteins containing β9, β10, or β10- β9 tNLuc tag C-terminally fused to protein G (C2 domain) and His tag were expressed in BL21-gold (DE3) *E. coli* bacteria. The proteins were purified using Nickel Sepharose according to the manufacturer’s instruction. The proteins were diluted at varied concentrations, mixed with Δ11S protein in a buffer containing Tris pH 7.5 20 mM, EDTA 2 mM, NaCl 25 mM, TCEP 0.5 mM, Tween20 0.1%, albumin 0.05%, and incubated for 3 h. Furimazine was then added in the mixture followed by luminescence measurements. Final concentration of Δ11S is 250 pM and furimazine is 25 μM. Kd of Δ11S with each tag was calculated through fitting the data to a model of specific binding with Hill slope using GraphPad.

### SIMPL2 assay

Culture medium of tested cells in 384-well plate was aspirated. An aliquot (50 μl) of reaction mixture containing Δ11S (0.4 μM) and furimazine (50 μM) in reaction buffer (Tris pH 7.5 20 mM, EDTA 1.2 mM, TCEP 0.24 mM, NaCl 30 mM, NP40 0.15%) was added into each well. After 1.5-h incubation at room temperature, luminescence was read in a luminescence microplate reader with integration time of one second.

### SIMPL2 assay for benchmark reference

HEK293 cells were seeded in 384-well plates with 5000 cells/well. SIMPL2 plasmids of genes under investigation were transfected into the cells in triplicates using PEI. Two genes (B and P) in SIMPL2 constructs (B-β10-IN and IC-β9-P) were co-transfected to measure the interaction within the corresponding protein pair. In parallel, each individual gene was co-transfected with a corresponding IN/IC handle to roughly measure the expression level. After 24 h, doxycycline (0.5 μg/ml final concentration) was added for 16 h incubation. The samples were subject to SIMPL2 assay. Comparison of SIMPL2 interaction signals was performed in three ways as follow. (1) Raw signals. (2) Normalized signals. Here the interaction signals were normalized (divided) by protein expression estimated using IN/IC handle. (3) Data transformed based on RRS distribution. For this, the linearity of SIMPL2 signals (in logarithmic scale) of RRS with their corresponding protein expression, in term of the product of two involved proteins (in logarithmic scale), was verified using simple linear regression. The derived linear equation (*y* = *α* + *βx*) of this regression was used to transform SIMPL2 signals of both RRS and PRS pairs using the formula *S*_*transformed*_ = *S*_*raw*_*- α - βS*_*BP*_. in which S_transformed_ is the interaction signal after transformation, S_raw_ is the raw interaction signal, S_BP_ is the product of B and P expressions, and all terms are in logarithmic scale.

### SIMPL2 assay for characterizing PPI modulators

Stable cells of target protein pair were created using Cas9 CRISPR. To characterize PPI modulators, the cells were seeded in 384 well plate (10,000 cells/well) and incubated for 5 h. Subsequently, they were treated with doxycycline (0.5 μg/ml) and tested compound for 16 h (sotorasib, AG1478, afatinib, selumetinib or RO-5963). In some experiments, the cells were treated with doxycycline and compound such as BI2865 for 5 h. Average signal of wells without either deoxycycline or compound treatment was used as blank or ‘floor’ signal (S_F_). The average signal of wells with deoxycycline treatment but without compound treatment was used as maximal interaction or ‘ceiling’ (S_C_). SIMPL2 reading of each sample (S_sample_) was normalized according to the formula S^*^_sample_ = *(S*_*sample*_*– S*_*F*_*)/(S*_*C*_*– S*_*F*_*) x 100%*, in which S^*^_sample_ stands for normalized signal. The normalized data of inhibitor experiments were fitted into a four-parameter inhibitor-response model. For PROTAC characterization, the cells were pretreated with doxycycline (1 μg/ml) for 16 h followed by ARV-825 treatment for 3 h in absence or present of MG132 (1 μM).

### NanoBiT assay

Stable cells of target protein pair were created using Cas9 CRISPR. The cells were grown in 396-well plate with OptiMEM medium containing 5% FBS and doxycycline (0.5 μg/ml) for 16 h, followed by treatment of rapamycin for 2 h, BI2865 for 5 h or ARV-825 for 3 h. Furimazine was then added to the final concentration of 31.25 μM. After 30 min, signals were read in a luminescence microplate reader.

### Statistics

Curve fitting to various mathematical models, receiver operating characteristic analysis and Student’s *t* test were performed using GraphPad. Blinding was not applied in this study.

## Supplementary information

Appendix

Peer Review File

Source data Fig. 1

Source data Fig. 2

Source data Fig. 3

Source data Fig. 4

Source data EV Figure

Expanded View Figures

## Data Availability

All data generated or analysed during this study are included in the source datasets of this published article. This study includes no data deposited in external repositories. The source data of this paper are collected in the following database record: biostudies:S-SCDT-10_1038-S44320-024-00081-2.

## References

[CR1] Arkin MMR, Wells JA (2004) Small-molecule inhibitors of protein-protein interactions: progressing towards the dream. Nat Rev Drug Discov 3:301–317. 10.1038/nrd1343 15060526 10.1038/nrd1343

[CR2] Arkin MR, Tang Y, Wells JA (2014) Small-molecule inhibitors of protein-protein interactions: progressing toward the reality. Chem Biol 21:1102–1114. 10.1016/j.chembiol.2014.09.001 25237857 10.1016/j.chembiol.2014.09.001 PMC4179228

[CR3] Arnold C (2023) Inside the nascent industry of AI-designed drugs. Nat Med 29:1292–1295. 10.1038/s41591-023-02361-0 37264208 10.1038/s41591-023-02361-0

[CR4] Banaszynski LA, Liu CW, Wandless TJ (2005) Characterization of the FKBP-rapamycin-FRB ternary complex. J Am Chem Soc 127:4715–4721 15796538 10.1021/ja043277y

[CR5] Benleulmi-Chaachoua A, Chen L, Sokolina K, Wong V, Jurisica I, Emerit MB, Darmon M, Espin A, Stagljar I, Tafelmeyer P et al (2016) Protein interactome mining defines melatonin MT1 receptors as integral component of presynaptic protein complexes of neurons. J Pineal Res 60:95–108 26514267 10.1111/jpi.12294

[CR6] Braun P, Tasan M, Dreze M, Barrios-Rodiles M, Lemmens I, Yu H, Sahalie JM, Murray RR, Roncari L, de Smet AS et al (2009) An experimentally derived confidence score for binary protein-protein interactions. Nat Methods 6:91–97 19060903 10.1038/nmeth.1281 PMC2976677

[CR7] Canon J, Rex K, Saiki AY, Mohr C, Cooke K, Bagal D, Gaida K, Holt T, Knutson CG, Koppada N et al (2019) The clinical KRAS(G12C) inhibitor AMG 510 drives anti-tumour immunity. Nature 575:217–223 31666701 10.1038/s41586-019-1694-1

[CR8] Choi SG, Olivet J, Cassonnet P, Vidalain PO, Luck K, Lambourne L, Spirohn K, Lemmens I, Dos Santos M, Demeret C et al (2019) Maximizing binary interactome mapping with a minimal number of assays. Nat Commun 10:3907 31467278 10.1038/s41467-019-11809-2 PMC6715725

[CR9] Dixon AS, Kim SJ, Baumgartner BK, Krippner S, Owen SC (2017) A tri-part protein complementation system using antibody-small peptide fusions enables homogeneous immunoassays. Sci Rep 7:8186 28811487 10.1038/s41598-017-07569-y PMC5557857

[CR10] Dixon AS, Schwinn MK, Hall MP, Zimmerman K, Otto P, Lubben TH, Butler BL, Binkowski BF, Machleidt T, Kirkland TA et al (2016) NanoLuc complementation reporter optimized for accurate measurement of protein interactions in cells. ACS Chem Biol 11:400–408 26569370 10.1021/acschembio.5b00753

[CR11] Graves B, Thompson T, Xia M, Janson C, Lukacs C, Deo D, Di Lello P, Fry D, Garvie C, Huang KS et al (2012) Activation of the p53 pathway by small-molecule-induced MDM2 and MDMX dimerization. Proc Natl Acad Sci USA 109:11788–11793 22745160 10.1073/pnas.1203789109 PMC3406834

[CR12] Grozavu I, Stuart S, Lyakisheva A, Yao Z, Pathmanathan S, Ohh M, Stagljar I (2022) D154Q mutation does not alter KRAS dimerization. J Mol Biol 434:167392 34896362 10.1016/j.jmb.2021.167392

[CR13] Hall MP, Kincaid VA, Jost EA, Smith TP, Hurst R, Forsyth SK, Fitzgerald C, Ressler VT, Zimmermann K, Lazar D et al (2021) Toward a point-of-need bioluminescence-based immunoassay utilizing a complete shelf-stable reagent. Anal Chem 93:5177–5184 33730483 10.1021/acs.analchem.0c05074

[CR14] Hall MP, Unch J, Binkowski BF, Valley MP, Butler BL, Wood MG, Otto P, Zimmerman K, Vidugiris G, MacHleidt T et al (2012) Engineered luciferase reporter from a deep sea shrimp utilizing a novel imidazopyrazinone substrate. ACS Chem Biol 7:1848–1857 22894855 10.1021/cb3002478 PMC3501149

[CR15] Hirano T, Yasuda H, Tani T, Hamamoto J, Oashi A, Ishioka K, Arai D, Nukaga S, Miyawaki M, Kawada I et al (2015) In vitro modeling to determine mutation specificity of EGFR tyrosine kinase inhibitors against clinically relevant EGFR mutants in non-small-cell lung cancer. Oncotarget 6:38789–38803 26515464 10.18632/oncotarget.5887 PMC4770737

[CR16] Kim D, Herdeis L, Rudolph D, Zhao Y, Böttcher J, Vides A, Ayala-Santos CI, Pourfarjam Y, Cuevas-Navarro A, Xue JY et al (2023) Pan-KRAS inhibitor disables oncogenic signalling and tumour growth. Nature 619:160–166 37258666 10.1038/s41586-023-06123-3 PMC10322706

[CR17] Kim SJ, Dixon AS, Adamovich PC, Robinson PD, Owen SC (2021) Homogeneous immunoassay using a tri-part split-luciferase for rapid quantification of anti-TNF therapeutic antibodies. ACS Sens 6:1807–1814 34010570 10.1021/acssensors.0c02642

[CR18] Kim SJ, Yao Z, Marsh MC, Eckert DM, Kay MS, Lyakisheva A, Pasic M, Bansal A, Birnboim C, Jha P et al (2022) Homogeneous surrogate virus neutralization assay to rapidly assess neutralization activity of anti-SARS-CoV-2 antibodies. Nat Commun 13:3716 35778399 10.1038/s41467-022-31300-9 PMC9249905

[CR19] Li D, Ambrogio L, Shimamura T, Kubo S, Takahashi M, Chirieac LR, Padera RF, Shapiro GI, Baum A, Himmelsbach F et al (2008) BIBW2992, an irreversible EGFR/HER2 inhibitor highly effective in preclinical lung cancer models. Oncogene 27:4702–4711 18408761 10.1038/onc.2008.109 PMC2748240

[CR20] Lim SH, Snider J, Birimberg‐Schwartz L, Ip W, Serralha JC, Botelho HM, Lopes‐Pacheco M, Pinto MC, Moutaoufik MT, Zilocchi M et al (2022) CFTR interactome mapping using the mammalian membrane two‐hybrid high‐throughput screening system. Mol Syst Biol 18:e10629 35156780 10.15252/msb.202110629 PMC8842165

[CR21] Liu X, Ciulli A (2023) Proximity-based modalities for biology and medicine. ACS Cent Sci 9:1269–1284. 10.1021/acscentsci.3c00395 37521793 10.1021/acscentsci.3c00395 PMC10375889

[CR22] Lopes JP, Morató X, Souza C, Pinhal C, Machado NJ, Canas PM, Silva HB, Stagljar I, Gandía J, Fernández-Dueñas V et al (2015) The role of Parkinson’s disease-associated receptor GPR37 in the hippocampus: functional interplay with the adenosinergic system. J Neurochem 134:135–146 25824528 10.1111/jnc.13109

[CR23] Lu H, Zhou Q, He J, Jiang Z, Peng C, Tong R, Shi J (2020) Recent advances in the development of protein–protein interactions modulators: mechanisms and clinical trials. Signal Transduct Target Ther 5. 10.1038/s41392-020-00315-3 10.1038/s41392-020-00315-3 PMC7511340 32968059

[CR24] Lu J, Qian Y, Altieri M, Dong H, Wang J, Raina K, Hines J, Winkler JD, Crew AP, Coleman K et al (2015) Hijacking the E3 ubiquitin ligase cereblon to efficiently target BRD4. Chem Biol 22:755–763 26051217 10.1016/j.chembiol.2015.05.009 PMC4475452

[CR25] Mock M, Edavettal S, Langmead C, Russell A (2023) AI can help to speed up drug discovery-but only if we give it the right data. Nature 621:467–470 37726439 10.1038/d41586-023-02896-9

[CR26] Neklesa TK, Winkler JD, Crews CM (2017) Targeted protein degradation by PROTACs. Pharm Ther 174:138–144. 10.1016/j.pharmthera.2017.02.027 10.1016/j.pharmthera.2017.02.027 28223226

[CR27] Ohmuro-Matsuyama Y, Ueda H (2018) Homogeneous noncompetitive luminescent immunodetection of small molecules by ternary protein fragment complementation. Anal Chem 90:3001–3004 29446920 10.1021/acs.analchem.7b05140

[CR28] Oliayi M, Emamzadeh R, Rastegar M, Nazari M (2023) Tri-part NanoLuc as a new split technology with potential applications in chemical biology: a mini-review. Anal Methods 15:3924–3931. 10.1039/d3ay00512g 37545367 10.1039/d3ay00512g

[CR29] Pathmanathan S, Grozavu I, Lyakisheva A, Stagljar I (2022) Drugging the undruggable proteins in cancer: a systems biology approach. Curr Opin Chem Biol 66. 10.1016/j.cbpa.2021.07.004 10.1016/j.cbpa.2021.07.004 34426091

[CR30] Pettersson M, Crews CM (2019) PROteolysis TArgeting Chimeras (PROTACs)—past, present and future. Drug Discov Today Technol 31:15–27. 10.1016/j.ddtec.2019.01.002 31200855 10.1016/j.ddtec.2019.01.002 PMC6578591

[CR31] Ran FA, Hsu PD, Wright J, Agarwala V, Scott DA, Zhang F (2013) Genome engineering using the CRISPR-Cas9 system. Nat Protoc 8:2281–2308 24157548 10.1038/nprot.2013.143 PMC3969860

[CR32] Rizzolo K, Huen J, Kumar A, Phanse S, Vlasblom J, Kakihara Y, Zeineddine HA, Minic Z, Snider J, Wang W et al (2017) Features of the chaperone cellular network revealed through systematic interaction mapping. Cell Rep 20:2735–2748 28903051 10.1016/j.celrep.2017.08.074

[CR33] Roberts AW, Stilgenbauer S, Seymour JF, Huang DCS (2017) Venetoclax in patients with previously treated chronic lymphocytic leukemia. Clin Cancer Res 23:4527–4534 28100580 10.1158/1078-0432.CCR-16-0955

[CR34] Saraon P, Snider J, Kalaidzidis Y, Wybenga-Groot LE, Weiss K, Rai A, Radulovich N, Drecun L, Vučković N, Vučetić A et al (2020) A drug discovery platform to identify compounds that inhibit EGFR triple mutants. Nat Chem Biol 16:577–586 32094923 10.1038/s41589-020-0484-2 PMC8123931

[CR35] Schreiber SL (2021) The rise of molecular glues. Cell 184:3–9 33417864 10.1016/j.cell.2020.12.020

[CR36] Scott DE, Bayly AR, Abell C, Skidmore J (2016) Small molecules, big targets: drug discovery faces the protein-protein interaction challenge. Nat Rev Drug Discov 15:533–550. 10.1038/nrd.2016.29 27050677 10.1038/nrd.2016.29

[CR37] Snider J, Kotlyar M, Saraon P, Yao Z, Jurisica I, Stagljar I (2015) Fundamentals of protein interaction network mapping. Mol Syst Biol 11:848 26681426 10.15252/msb.20156351 PMC4704491

[CR38] Trepte P, Kruse S, Kostova S, Hoffmann S, Buntru A, Tempelmeier A, Secker C, Diez L, Schulz A, Klockmeier K et al (2018) LuTHy: a double‐readout bioluminescence‐based two‐hybrid technology for quantitative mapping of protein–protein interactions in mammalian cells. Mol Syst Biol 14:e8071 29997244 10.15252/msb.20178071 PMC6039870

[CR39] Trepte P, Secker C, Olivet J, Blavier J, Kostova S, Maseko SB, Minia I, Silva Ramos E, Cassonnet P, Golusik S et al (2024) AI-guided pipeline for protein–protein interaction drug discovery identifies a SARS-CoV-2 inhibitor. Mol Syst Biol 20:428–457 38467836 10.1038/s44320-024-00019-8 PMC10987651

[CR40] Vakili MG, Gorgulla C, Nigam A, Bezrukov D, Varoli D, Aliper A, Polykovsky D, Das KMP, Snider J, Lyakisheva A et al (2024) Quantum computing-enhanced algorithm unveils novel inhibitors for KRAS. Preprint at https://arxiv.org/abs/2402.08210

[CR41] Venkatesan K, Rual JF, Vazquez A, Stelzl U, Lemmens I, Hirozane-Kishikawa T, Hao T, Zenkner M, Xin X, Goh K, Il et al (2009) An empirical framework for binary interactome mapping. Nat Methods 6:83–90 19060904 10.1038/nmeth.1280 PMC2872561

[CR42] Yaish P, Gazit A, Gilon C, Levitzki A (1988) Blocking of EGF-dependent cell proliferation by EGF receptor kinase inhibitors. Science 242:933–935 3263702 10.1126/science.3263702

[CR43] Yao Z, Aboualizadeh F, Kroll J, Akula I, Snider J, Lyakisheva A, Tang P, Kotlyar M, Jurisica I, Boxem M et al (2020) Split intein-mediated protein ligation for detecting protein-protein interactions and their inhibition. Nat Commun 11:2440 32415080 10.1038/s41467-020-16299-1 PMC7229206

[CR44] Yao Z, Darowski K, St-denis N, Babu M, Gingras A, Stagljar I, Interactome TKP (2017) A global analysis of the receptor tyrosine kinase- resource A global analysis of the receptor. Mol Cell 65:347–360 28065597 10.1016/j.molcel.2016.12.004 PMC5663465

[CR45] Yao Z, Drecun L, Aboualizadeh F, Kim SJ, Li Z, Wood H, Valcourt EJ, Manguiat K, Plenderleith S, Yip L et al (2021) A homogeneous split-luciferase assay for rapid and sensitive detection of anti-SARS CoV-2 antibodies. Nat Commun 12:1806 33753733 10.1038/s41467-021-22102-6 PMC7985487

[CR46] Yao Z, Petschnigg J, Ketteler R, Stagljar I (2015) Application guide for omics approaches to cell signaling. Nat Chem Biol 11:387–397 25978996 10.1038/nchembio.1809

