# Supplementary material for: A split intein and split luciferase-coupled system for detecting protein-protein interactions
Source: Mol Syst Biol. 2024 Dec 12;21(2):1. doi: 10.1038/s44320-024-00081-2 (PMC11791039; doi:10.1038/s44320-024-00081-2)
Supplement: Supplementary file 1 — Appendix [file 44320_2024_81_MOESM1_ESM.pdf]

Appendix for

A Split Intein and Split Luciferase-Coupled System for Detecting Protein-Protein Interactions

Appendix file:

Page 1: **Appendix Figure S1**. DNA constructs used in this study.

A

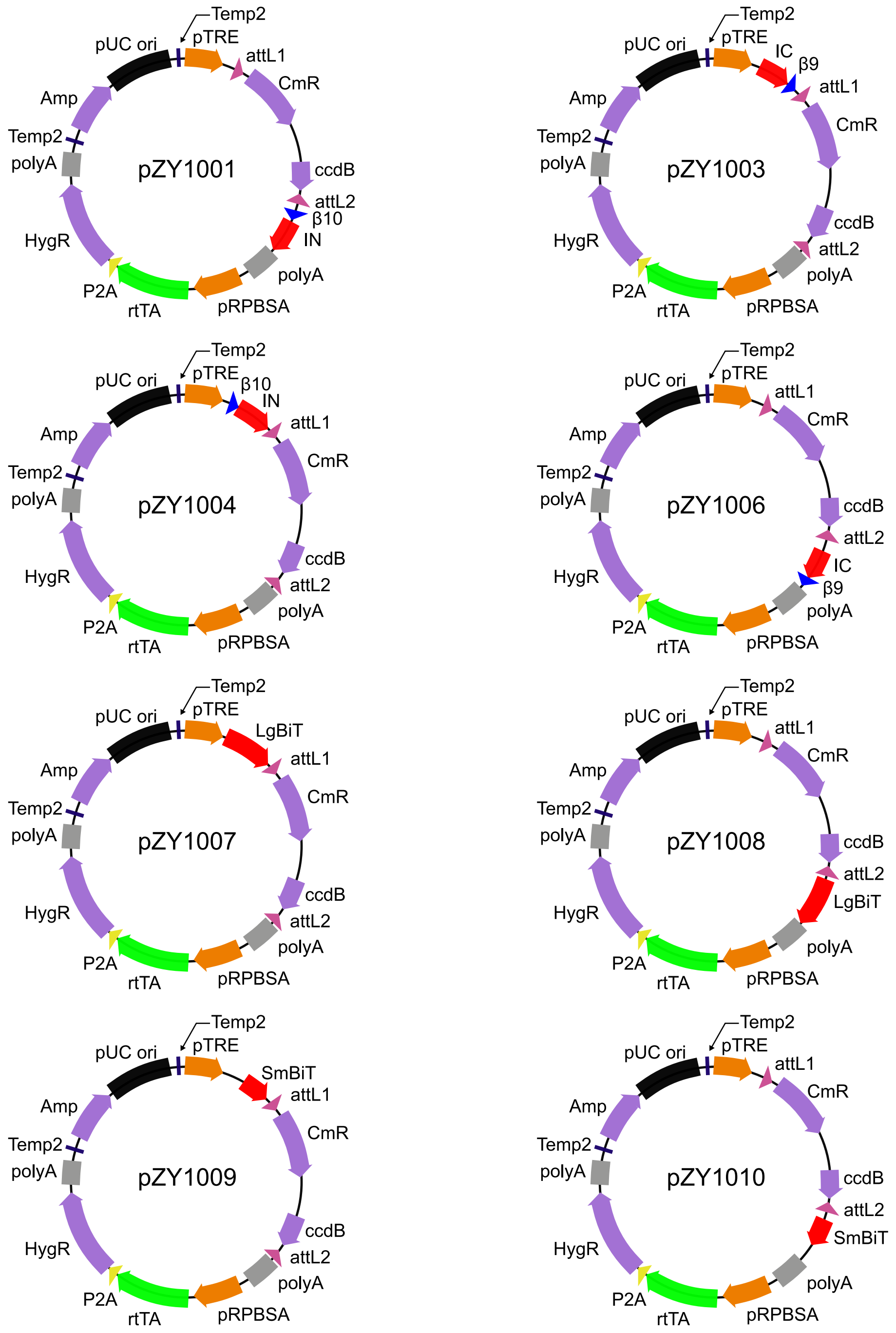

B

Gene	Vector	Assay	Experiment
FRB	pZY1001	SIMPL2	Fig. 1D, Fig. EV1C
FKBP1A	pZY1006	SIMPL2	Fig. 1D, Fig. EV1C
FKBP1A	pZY1003	SIMPL2	Fig. EV1B
FRB	pZY1008	NanoBiT	Fig. 1D
FKBP1A	pZY1010	NanoBiT	Fig. 1D
KRAS	pZY1003	SIMPL2	Fig. 3A, Fig. EV3A
RBD	pZY1004	SIMPL2	Fig. 3A, Fig. EV3A
KRAS	pZY1009	NanoBiT	Fig. EV3A
RBD	pZY1007	NanoBiT	Fig. EV3A
EGFR	pZY1001	SIMPL2	Fig. 3B-E, Fig. EV3B
SHC1	pZY1003	SIMPL2	Fig. 3B-E, Fig. EV3B
MDM2	pZY1001	SIMPL2	Fig. 4A
MDM4	pZY1003	SIMPL2	Fig. 4A
CRBN	pZY1004	SIMPL2	Fig. 4B, Fig. EV4A-B
BRD4	pZY1003	SIMPL2	Fig. 4B, Fig. EV4A-B
CRBN	pZY1009	NanoBiT	Fig. EV4B
BRD4	pZY1007	NanoBiT	Fig. EV4B

Appendix figure S1. DNA constructs used in this study.
A, Plasmid vectors created for SIMPL2 and NanoBiT assays.
B, Various plasmid constructs used in the study.
