# Supplementary material for: A split intein and split luciferase-coupled system for detecting protein-protein interactions
Source: Mol Syst Biol. 2024 Dec 12;21(2):1. doi: 10.1038/s44320-024-00081-2 (PMC11791039; doi:10.1038/s44320-024-00081-2)
Supplement: Supplementary file 8 — Expanded View Figures [file 44320_2024_81_MOESM8_ESM.pdf]

Expanded View Figures

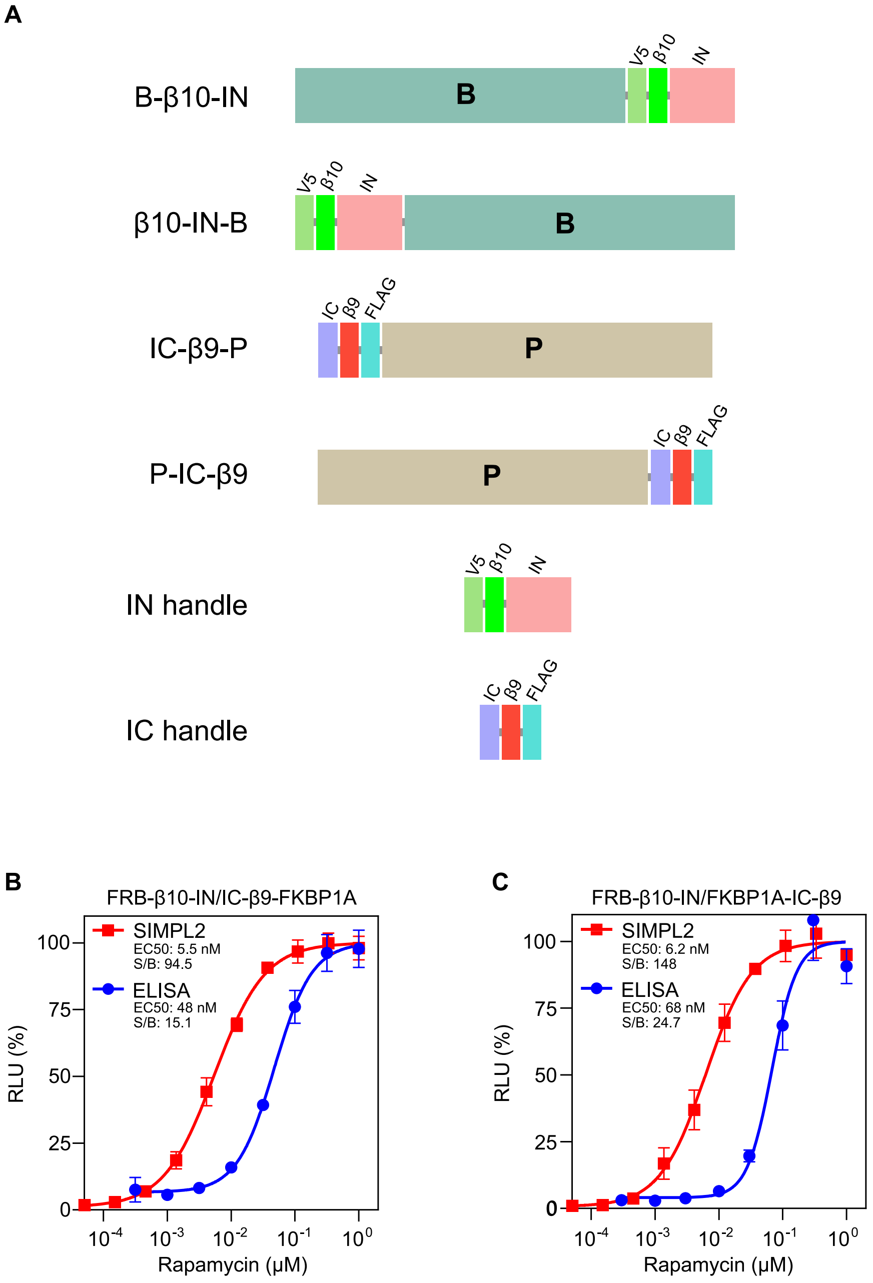

Figure EV1. SIMPL2 constructs.

(**A**) A protein of interest "B" can be tagged with β10-IN on either its N- or C-terminus. Similarly, a second protein of interest "P" can be tagged with IC-β9 on either terminus. IN/IC handles are used for estimating protein expression. (**B**, **C**) Comparison of the SIMPL2 and SIMPL-ELISA assays performed using rapamycin-mediated FRB/FKBP1A interaction. Cells expressing FRB-β10-IN and IC-β9-FKBP1A (**B**) or FRB-β10-IN and FKBP1A-IC-β9 (**C**) were subject to rapamycin treatment with varied concentrations for 2 h followed by measurement of SIMPL2 signal. Data of SIMPL-ELISA with corresponding configuration combinations were extracted from previous study (*Nat Commun* (2020) 11:2440). The data were fit to a four-parameter agonist-response model, with corresponding curves shown. The raw data are normalized with corresponding maximal signals and presented as mean ± s.d. with $n = 4$ biological replicates for both SIMPL2 and SIMPL-ELISA in (**B**), $n = 3$ biological replicates for SIMPL2 and $n = 4$ biological replicates for SIMPL-ELISA in (**C**). Signal to background (S/B) ratios were calculated based on the derived models. Source data are available online for this figure.

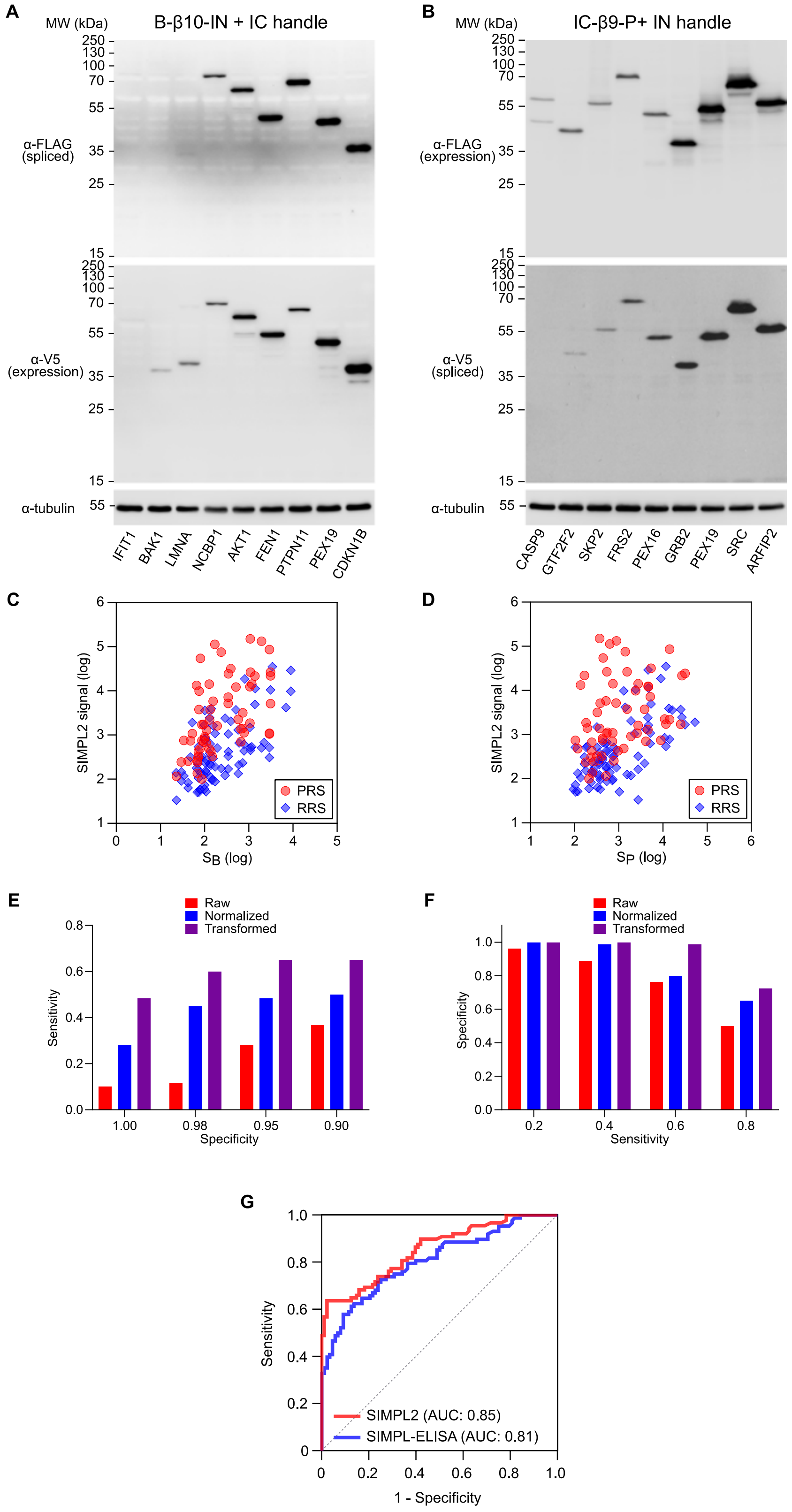
A
MW (kDa)
B-β10-IN + IC handle
α-FLAG (spliced)
α-V5 (expression)
α-tubulin
IFIT1
BAK1
LMNA
NCBP1
AKT1
FEN1
PTPN11
PEX19
CDKN1B
B
IC-β9-P+ IN handle
α-FLAG (expression)
α-V5 (spliced)
CASP9
GTF2F2
SKP2
FRS2
PEX16
GRB2
PEX19
SRC
ARFIP2
C
SIMPL2 signal (log)
SB (log)
PRS
RRS
D
Sp (log)
E
Raw
Normalized
Transformed
Sensitivity
Specificity
F
G
SIMPL2 (AUC: 0.85)
SIMPL-ELISA (AUC: 0.81)
1 - Specificity

Figure EV2. Behavior of reference set in the SIMPL2 system.

(**A**) β10-IN-tagged Protein 'B' constructs were co-expressed with IC handle in HEK 293T cells and the cell lysates were subjected to Western blot analysis. The total expression level of B was detected using α-V5 antibody, while levels of spliced product caused by non-specific interaction with IC handle were detected using α-FLAG antibody. (**B**) IC-β9-tagged Protein 'P' constructs were co-expressed with IN handle, followed by western blot analysis using α-V5 antibody and α-FLAG antibody, to detect protein expression levels as described in (**A**). (**C**, **D**) Interaction signals of RRS and PRS in Fig. 2 were plotted against 'handle' signal correlated with B protein expression (S_B in logarithmic scale) (**C**) or P protein expression (S_P in logarithmic scale) (**D**). (**E**, **F**) Evaluation of different data processing methods. Based on the ROC curves obtained in Fig. 2B, E, H, sensitivities of different data processing methods at selected specificities (1.00, 0.98, 0.95 or 0.90) are shown in (**E**). Similarly, specificities of the methods at selected sensitivities (0.2, 0.4, 0.4 or 0.8) are shown in (**F**). (**G**) Comparison of SIMPL2 and SIMPL-ELISA. ROC curve of a SIMPL-ELISA assay with a PRS set ($n = 88$) and an RRS set ($n = 88$) (published in *Nat Commun* 11:2440) is compared with that from a SIMPL2 assay using the same reference sets. Source data are available online for this figure.

© The Author(s)

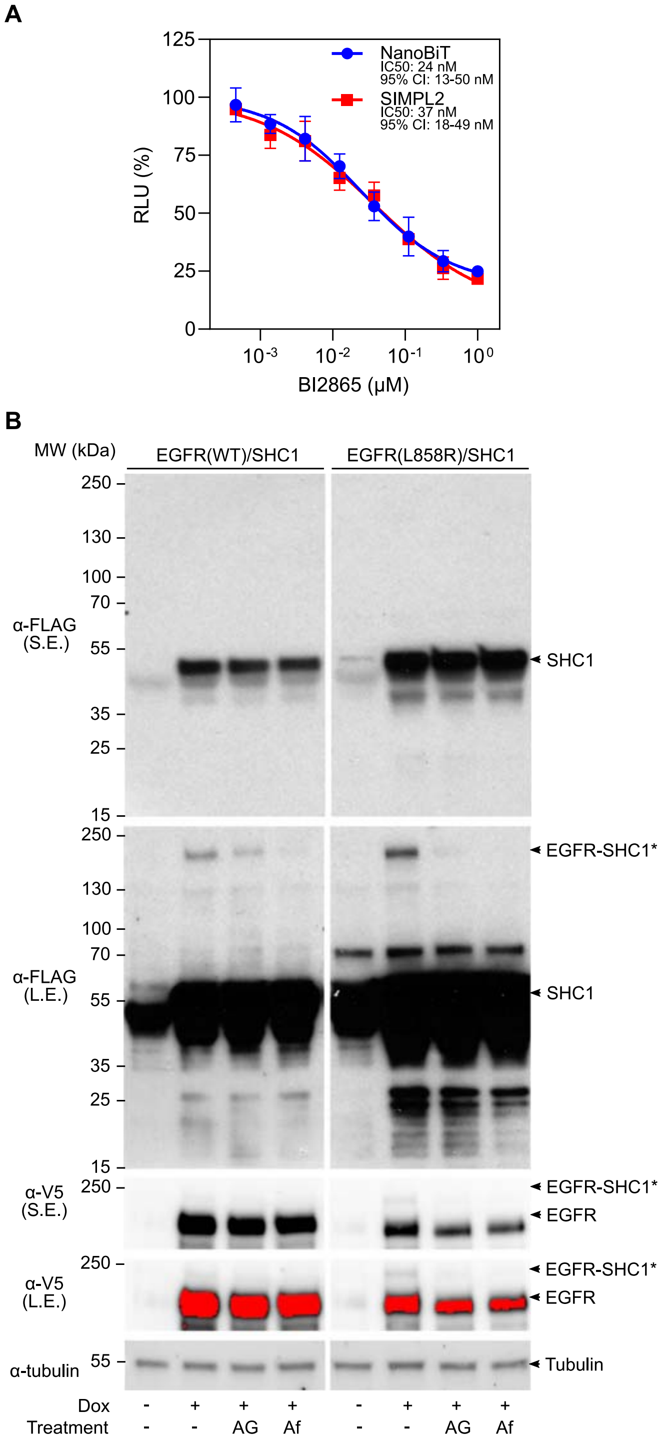
A
NanoBiT
IC50: 24 nM
95% CI: 13-50 nM
SIMPL2
IC50: 37 nM
95% CI: 18-49 nM
RLU (%)
BI2865 (μM)
B
MW (kDa)
EGFR(WT)/SHC1
EGFR(L858R)/SHC1
α-FLAG (S.E.)
α-FLAG (L.E.)
α-V5 (S.E.)
α-V5 (L.E.)
α-tubulin
SHC1
EGFR-SHC1*
EGFR
Tubulin
Dox
Treatment
AG
Af

 © The Author(s)

Figure EV3. Validation of SIMPL2 as method for detecting PPI inhibition.

(**A**) Comparison of SIMPL2 and NanoBiT in evaluating the KRAS inhibitor BI2865. SIMPL2 stable cells expressing IC-β9-KRAS(WT)/β10-IN-RBD were simultaneously treated with both doxycycline and BI2865 for 5 h at the indicated concentrations followed by luminescence measurement. In contrast, the expression of SmBiT-KRAS(WT)/LgBiT-RBD was induced for 16 h in NanoBiT stable cells, followed by BI2865 treatment. Data are presented as mean ± s.d. with $n = 4$ biological replicates and were fitted into a four-parameter inhibitor-response model. (**B**) The expression of EGFR (WT or L858R)-β10-IN and IC-β9-SHC1 in stable cells was induced with doxycycline plus treatment with AG1478 (AG, 100 nM) or afatinib (Af, 100 nM). After 16 h of treatment, the cells were lysed and subjected to western blot analysis. Both blots with short exposure (S.E.) and long exposure (L.E.) are presented. Interaction-induced splicing produced a fusion protein of EGFR-SHC1 (*). Source data are available online for this figure.

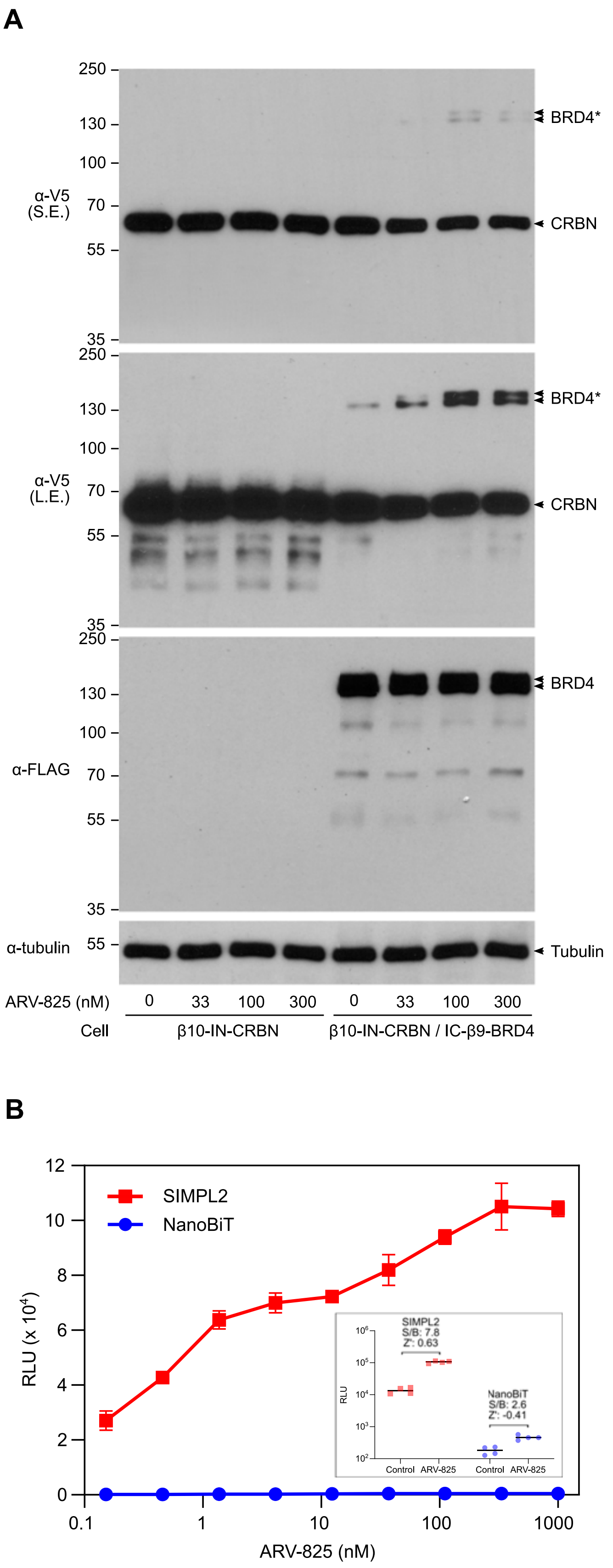
A
250
130
100
70
55
35
α-V5
(S.E.)
BRD4*
CRBN
α-V5
(L.E.)
BRD4*
CRBN
α-FLAG
BRD4
α-tubulin
Tubulin
ARV-825 (nM)
0
33
100
300
0
33
100
300
Cell
β10-IN-CRBN
β10-IN-CRBN / IC-β9-BRD4
B
SIMPL2
NanoBiT
RLU (x 10⁴)
12
10
8
6
4
2
0
0.1
1
10
100
1000
ARV-825 (nM)
SIMPL2
S/B: 7.8
Z': 0.63
NanoBiT
S/B: 2.6
Z': -0.41
RLU
Control
ARV-825
Control
ARV-825

 © The Author(s)

Figure EV4. Validation of SIMPL2 as method for detecting PROTACs using a Western blot readout.

(**A**) The expression of β10-IN-CRBN/IC-β9-BRD4 in stable cells was induced with doxycycline for 16 h followed by treatment with ARV-825 at the indicated concentrations for 3 h. The cells were lysed and subjected to Western blot analysis. Stable cells expressing only β10-IN-CRBN were used as a control. ARV-825 induced splicing allowed V5 tag transfer to BRD4, which presents a BRD4 band (*) in the V5 blot. (**B**) Comparison of SIMPL2 and NanoBiT in evaluating PROTAC. Stable cells expressing β10-IN-CRBN/IC-β9-BRD4 or SmBiT-CRBN/LgBiT-BRD4 were treated with ARV-825 at the indicated concentration for three hours followed by signal measurement. Data are presented as mean ± s.d. with $n = 4$ biological replicates. S/B signal/background ratio. Source data are available online for this figure.
