# Supplementary material for: A split intein and split luciferase-coupled system for detecting protein-protein interactions
Source: Mol Syst Biol. 2024 Dec 12;21(2):1. doi: 10.1038/s44320-024-00081-2 (PMC11791039; doi:10.1038/s44320-024-00081-2)
Supplement: Supplementary file 2 — Peer Review File [file 44320_2024_81_MOESM2_ESM.pdf]

molecular systems biology

A Split Intein and Split Luciferase-Coupled System for Detecting Protein-Protein Interactions

Igor Stagljar, Zhong Yao, Jiyoon Kim, Betty Geng, Jinkun Chen, Anna Lyakisheva, Jamie Snider, Marina Dimlić, Sanda Raić, and Victoria Wong

Corresponding author(s): Igor Stagljar (igor.stagljar@utoronto.ca) , Zhong Yao (zhong.yao@utoronto.ca)

Review Timeline:

Submission Date:	27th Mar 24
Editorial Decision:	17th May 24
Revision Received:	14th Oct 24
Editorial Decision:	13th Nov 24
Revision Received:	21st Nov 24
Accepted:	29th Nov 24

Editors: Maria Polychronidou and Poonam Bheda

Transaction Report:

(Note: With the exception of the correction of typographical or spelling errors that could be a source of ambiguity, letters and reports are not edited. Depending on transfer agreements, referee reports obtained elsewhere may or may not be included in this compilation. Referee reports are anonymous unless the Referee chooses to sign their reports.)

17th May 2024

Manuscript Number: MSB-2024-12338
Title: A Split Intein and Split Luciferase-Coupled System for Detecting Protein-Protein Interactions

Dear Igor,

Thank you again for submitting your work to Molecular Systems Biology. We have now heard back from two of the three reviewers who agreed to evaluate your study. Unfortunately, after several reminders we did not receive a report from reviewer #1. In the interest of time, we have decided to proceed with making a decision based on the two available reports. As you will see below the reviewers acknowledge that the presented method is relevant for analysing protein-protein interactions. However, they raise several concerns, which we would invite you to address in a revision.

I think that the reviewers' recommendations are rather clear and I therefore see no need to repeat the comments listed below. Of note, reviewer #3 refers to the need to describe potential limitations of the presented approach in a more balanced manner and to better contextualise the benefits compared to existing approaches.

All issues raised by the reviewers would need to be satisfactorily addressed. As you may already know, our editorial policy allows in principle a single round of major revision. It is therefore essential to provide responses to the reviewers' comments that are as complete as possible. If you have any questions of if would like to discuss your revision plan with me, please feel free to get in touch.

On a more editorial level, we would ask you to address the following points:

- We would ask you to use the article type "Method".

- Please provide a .doc version of the manuscript text (including legends for main Figures and EV Figures) and individual production quality figure files for the main Figures and EV Figures (one file per figure).

- Please include 5 keywords.

- We have replaced Supplementary Information by the Expanded View (EV format). In this case (unless the number of EV figures becomes > 6 during revision), all additional figures can be provided as EV Figures. Please provide one file per EV Figure. Their legends should be included in the manuscript text. For detailed instructions regarding expanded view please refer to our Author Guidelines: .

- Please provide a "standfirst text" summarizing the study in one or two sentences (approximately 250 characters), three to four "bullet points" highlighting the main findings and a "synopsis image" (exactly 550px width and max 400px height, jpeg or png format) to highlight the paper on our homepage.

- All Materials and Methods need to be described in the main text. We would ask you to use 'Structured Methods', our new Materials and Methods format, which is mandatory for Methods and Articles with a strong methodological focus. According to this format, the Material and Methods section should include a Reagents and Tools Table (listing key reagents, experimental models, software and relevant equipment and including their sources and relevant identifiers) followed by a Methods and Protocols section in which we encourage the authors to describe their methods using a step-by-step protocol format with bullet points, to facilitate the adoption of the methodologies across labs. More information on how to adhere to this format as well as downloadable templates (.doc or .xls) for the Reagents and Tools Table can be found in our author guidelines: . An example of a Method paper with Structured Methods can be found here: .

- Please include a Data availability section describing how the data and code have been made available. This section needs to be formatted according to the example below:
The datasets and computer code produced in this study are available in the following databases:
- Chip-Seq data: Gene Expression Omnibus GSE46748 (https://www.ncbi.nlm.nih.gov/geo/query/acc.cgi?acc=GSE46748)
- Modeling computer scripts: GitHub (https://github.com/SysBioChalmers/GECKO/releases/tag/v1.0)
- [data type]: [full name of the resource] [accession number/identifier] ([doi or URL or identifiers.org/DATABASE:ACCESSION])

- For data quantification: please specify the name of the statistical test used to generate error bars and P values, the number (n) of independent experiments (specify technical or biological replicates) underlying each data point and the test used to calculate p-values in each figure legend. The figure legends should contain a basic description of n, P and the test applied. Graphs must include a description of the bars and the error bars (s.d., s.e.m.).

- Please include a "Disclosure & Competing Interests Statement" in the main text.

- The References should be formatted according to the Molecular Systems Biology reference style (i.e., ordered alphabetically and listing the first 10 authors followed by et al).

- When you resubmit your manuscript, please download our CHECKLIST (https://bit.ly/EMBOPressAuthorChecklist) and include the completed form in your submission.
Please note that the Author Checklist will be published alongside the paper as part of the transparent process (https://www.embopress.org/page/journal/17444292/authorguide#transparentprocess).

If you feel you can satisfactorily deal with these points and those listed by the referees, you may wish to submit a revised version of your manuscript. Please attach a covering letter giving details of the way in which you have handled each of the points raised by the referees. A revised manuscript will be once again subject to review and you probably understand that we can give you no guarantee at this stage that the eventual outcome will be favorable.

Kind regards,

Maria

Maria Polychronidou, PhD
Senior Editor
Molecular Systems Biology

We realize that it is difficult to revise to a specific deadline. In the interest of protecting the conceptual advance provided by the work, we recommend a revision within 3 months (15th Aug 2024). Please discuss the revision progress ahead of this time with the editor if you require more time to complete the revisions. Use the link below to submit your revision:

https://msb.msubmit.net/cgi-bin/main.plex

IMPORTANT: When you send your revision, we will require the following items:
1. the manuscript text in LaTeX, RTF or MS Word format
2. a letter with a detailed description of the changes made in response to the referees. Please specify clearly the exact places in the text (pages and paragraphs) where each change has been made in response to each specific comment given
3. three to four 'bullet points' highlighting the main findings of your study
4. a short 'blurb' text summarizing in two sentences the study (max. 250 characters)
5. a 'thumbnail image' (550px width and max 400px height, Illustrator, PowerPoint or jpeg format), which can be used as 'visual title' for the synopsis section of your paper.
6. Please include an author contributions statement after the Acknowledgements section (see https://www.embopress.org/page/journal/17444292/authorguide)
7. Please complete the CHECKLIST available at (https://bit.ly/EMBOPressAuthorChecklist).
Please note that the Author Checklist will be published alongside the paper as part of the transparent process (https://www.embopress.org/page/journal/17444292/authorguide#transparentprocess).
8. When assembling figures, please refer to our figure preparation guideline in order to ensure proper formatting and readability in print as well as on screen:
https://bit.ly/EMBOPressFigurePreparationGuideline
See also figure legend guidelines: https://www.embopress.org/page/journal/17444292/authorguide#figureformat
9. Please note that corresponding authors are required to supply an ORCID ID for their name upon submission of a revised manuscript (EMBO Press signed a joint statement to encourage ORCID adoption).
(https://www.embopress.org/page/journal/17444292/authorguide#editorialprocess)
Currently, our records indicate that the ORCID for your account is 0000-0002-5260-3327.

Please click the link below to modify this ORCID:
Link Not Available

10. At EMBO Press we ask authors to provide source data for the main manuscript figures. Our source data coordinator will contact you to discuss which figure panels we would need source data for and will also provide you with helpful tips on how to upload and organize the files.

As a matter of course, please make sure that you have correctly followed the instructions for authors as given on the submission website.

*** PLEASE NOTE *** As part of the EMBO Press transparent editorial process initiative (see our Editorial at https://dx.doi.org/10.1038/msb.2010.72), Molecular Systems Biology publishes online a Review Process File with each accepted manuscripts. This file will be published in conjunction with your paper and will include the anonymous referee reports, your point-by-point response and all pertinent correspondence relating to the manuscript. If you do NOT want this File to be published, please inform the editorial office at msb@embo.org within 14 days upon receipt of the present letter.

Reviewer #2:

Report on Yao et al. " A Split Intein and Split Luciferase-Coupled System for Detecting Protein-Protein Interactions"

Targeting interaction through glues, inhibitors and Protacs is one of the main most promising developments in pharmacological research currently.
The Split Intein-Mediated Protein Ligation of interaction pairs detection developed by Yao et al previously was based on V5/FLAG/GFP western blotting readouts requiring several configurations of the tagged proteins. Here they report a triple split-nanoLuc system that is high throughput compatible, in a sense that established PPIs can be assayed e.g. for inhibition at scale as opposed to a system that can screen a large search space for novel interactions. In short, interaction leads to the protein splicing and fusion of b8-b9 of nanoLuc and D11S is added with the substrate after cell permeabilization providing a highly specific luciferase signal with reduced background.
Notably some background splicing exist that can be measured with "handle" constructs and used for data processing, as the raw signals as such do not very well distinguish the set of PRS from RRS interactions. Normalizations refers to dividing by the product of background signal from the B and P handles, transformation refers to using the linear regression for flattening the dependency of the handle signal product. This was used to determine the assay sensitivity and gains to reach 0.48.
For the other experiments, the intein constructs are stably integrated in the cell lines and placed under Tet induction.
Ras-Raf is examined in the presence of cancer mutations and dose dependent inhibitor treatment (sotorasib) as well as the EGFR-Shc interaction in the presence of afatinib or AG1478.
In another application MDM2-MDM4 is glued with RO-5963 administered to the cells. Also the BRD4-CRBN recruitment via small molecules from the most famous PROTAC system can be well monitored under proteasomal inhibitory conditions.
This paper is very clearly written and a very clear essential presentation of the substantially improved method. I would indeed argue that the triple-nanoLUC system presented here advances the method from a low throughput specialized application to a well controlled and broadly applicable, quantitative method in PPI research. The benchmark is thorough and useful, specific examples provide excellent data on PPI inhibition. The assay was performed in 384 plate format and given well defined PPI pairs it would clearly enable a drug screening endeavor! It is an excellent, highly focused systems biology methods-study.

Points for consideration:
*) It is not fully discussed whether cells are lysed or only permeable, and whether the system would work in the lysate as well. This refers to drug treatment. How do we know when to add the drug? In the Ras-Raf experiment induction and addition of the drug happen together for 16h. How do we control for expression level changes upon drug treatment for 16h? For the glue experiments 3h small molecule treatment was used. Can the PPI be induced after permeabilization?
*) Because of the irreversibility of the splicing event, I would assume the complexes are removed from binding equilibrium. Can the authors discuss why then the dose dependent binding inhibition actually works so nicely?
*) Intrinsic splicing dependent signals vary depending on the construct. Are there protein constructs where the signal with the respective handle reaches signals so high that they need to be fully excluded from the analysis? This would be the analogy to autoactivation of certain proteins, observed in other types assays.
*) In the AUC analyses it says what the sensitivity is at the chosen cut off, but not what is the luminescence signal cut off actually.
*) Maybe the global screening strategy "Thus, we conclude that SIMPL2 is well suited for high throughput PPI discovery by screening large open reading frame (ORF) pools fused to IN or IC." is an overstatement, or needs more details. How would you sort out the binders from non-binders in a pooled experiment? The luciferase signal is not connected to DNA or so ...
*) ... of rapid experimental validation which does not bottleneck the discovery process ...Can "bottleneck" be used as a verb like in this sentence?

Reviewer #3:

In this paper, Yao et al. present SIMPL2. an evolution of their previously described SIMPL (Split Intein-Mediated Protein Ligation) assay for detecting protein-protein interactions (PPI). The original SIMPL was based on the fusion of a pair of proteins to be tested to complementary intein fragments. The fragments had been optimized to minimize self-assembly and each fusion protein also contains a small detection tag. If the two proteins of interest interact together, their bring the two intein fragments in

close proximity thereby allowing them to re-assemble in an active intein. This leads to the ligation of the two proteins of interest, each of them bringing its detection tags. PPI are then detected by western blot analysis of the two tags or by ELISA.
In pSIMPL2, the system is combined to a tripartite split nanoluc luciferase detection system: two nanoluc peptides are fused to the split-intein fusion proteins so that upon successful intein-mediated protein ligation they are fused together. An otherwise inactive nanoluc fragment can regain activity upon binding to the fused nanoluc peptide, hence provide a sensitive and easy read-out for PPI.

Altogether the authors provide convincing evidence that SIMPL2 is an upgrade compared to SIMPL with higher sensitivity and much easier and cost-effective handling. The data they use to make their point were generated with well-designed experiments.
My main criticisms to the manuscript are the following:
- the repeating tendency of the authors to over emphasize the positive aspects of SIMPL2 while superficially discussing its limitations.
- The lack of evidence that SIMPL2 brings any added value compared to a split-nanoluc (NanoBiT) assay. This one is my main criticism as it considerably limits the general interest for this manuscript.

Below is a list of more detailed comments:

1) It is claimed that SIMPL2 demonstrates "an improvement in both sensitivity and specificity over the original SIMPL assay" (abstract and introduction on page 4). However there is only a single direct comparison of SIMPL vs SIMPL2 provided on Fig. 1D. It does suggest improved sensitivity of SIMPL2 but does not provide any evidence of improved specificity. No other data in the manuscript allow making this latter point (a strategy for data transformation is presented on Fig.2 that leads to a more specific identification of hits within the SIMPL2 framework but whether SIMPL2 is more specific than SIMPL has not been addressed).

2) It appears to me that a split-nanoluc assay (NanoBiT) would be a better choice than SIMPL2 in most situations: it is an easier setup (two fusion proteins, does not require cell permeabilization and apart from furimazine no additional reagent), is more sensitive (for the rapamycin7FRB/FKBP system Promega shows an EC50 of 1.7 nm for rapamycin measured with NanoBiT vs. 5.5 nm measured with SIMPL2 in the present study), and has the huge advantage of being reversible (and hence allows studying dynamic PPI, also with wash out and/or competition experiments). Can the authors provide experimental evidence that there any reason/specific situation to use SIMPL2 rather than NanoBiT?

The general tone and argumentation of the manuscript presents SIMPL2 almost as the panacea when studying PPI with all other available assays described as replete with limitations. I acknowledge that the authors are excited about their new method but they should try not be too carried out by enthusiasm and thereby forget to guide the non-seasoned reader in avoiding potential pitfalls attached to the assay. The next comments point potential limitations of the assay that would need to be discussed by the authors:

3) The assay suffers from its non-reversibility combined with the background splicing activity (shown on Fig. EV2) of the intein fragments. This imposes limitations that are not clearly stated:
a. SIMPL2 can be used to test PPI modulators but only if the modulator is applied to the cells before the tested proteins are expressed (otherwise irreversible splicing will have already occurred). This means the suitable setups are virtually only transient transfections or inducible stable cell lines.
b. Non-reversibility + low, but detectable, background protein splicing activity also implies that tagging constitutively expressed proteins (e.g. through genome editing or even transient expression over a long time) is most probably not possible: this is not suitable in any case for studying PPI modulators (see 3.a.) and will lead to huge background signal for any pair of intein-tagged proteins (constitutive expression over days will necessarily lead to the accumulation of intein-mediated ligated products over time due to the background activity).
c. It appears (Fig. EV2C & D) that there is a significant number of PRS for which the signal of the negative control (SB or SP) is higher than the SIMPL2 signal, which means that for those pair of proteins with validated binary interaction, coupling one of the two with an IC or IN handle yields a higher signal than when measuring the SIMPL2 constructs. I am aware that with the data transformation strategy presented on Fig. 2, the authors provide a strategy to define a cutoff that will lead to the identification of real positive hits most of the time. However this is feasible in a HTS set up but for those testing a single pair of proteins this strategy will not be applicable and it means that performing both IC and IN handle controls is critical to ensure the SIMPL2 signals are specific. This is not enough emphasized in the conclusion of the article.
d. Looking at the data it seems to me that the expression levels of the fusion proteins must be kept under a certain threshold: on Fig. 2C, F, I it looks like above a SBP (log) of approx. 6.5 the background signal is just too high to allow identify any real interaction. Can the authors comment on this, does this threshold clearly corresponds to an overexpression or does it mean that above a certain physiological expression level SIMPL2 is just not applicable?

4) Fig. 3A: in contrast to what the main text claims there is an inhibition (approx. 40-50%) observed at high sotorasib concentrations when the negative control HRAS/RBD and EGF/SHC1 cells were tested. This needs a comment.

5) Page 10: the rational for testing WT and L858R variant of EGFR is not explained. Was it expected that the two tested

compounds would inhibit the oncogenic variant more efficiently?

6) For all the IC50 measured, how do they compare with previously published data?

Minor points:
- In the abstract, for the sake of precision please write : "... for detecting binary PPI via..." instead of : "... for detecting PPI via..."

- Page 4: "providing a means to help meet the current demand for new therapeutic approaches to treat serious disease." This far-fetched statement is really not needed, the method is nice enough by itself that it does not need to be pimped with the extraordinary claim that it will help cure serious disease. It is a nice addition indeed to the toolbox of methods for studying PPIs, but how it will be used for in the future and what it will help to achieve is anyone's guess.
- Page 7. In the sentence "However, if the proteins under study are sufficiently stable, even trace levels of background, non-specifically spliced product could accumulate..." I guess the authors meant "...even trace with levels of background..."
- The explanations for the PRS and RRS sets are limited to the sentence "We next used reference PPIs to evaluate the performance of the SIMPL2 system. hsPRS-v2 (n=60) was used as the positive reference set (PRS) in this study30. The random reference set (RRS) contains 80 protein pairs with baits and preys selected from hsPRS-v1" on page 7. Please consider changing the wording (this study: does it refer to the present study or to ref. 30?) add more explanations for the non-seasoned reader: without knowing Ref. 30, the reader has no idea what are hsPRS-v2 and hsPRS-v1 and what was the rational to construct the RRS set from v1 rather than v2. At the present these few lines are too quick on the explanations making it hard to follow.
- Page 8: the wording "by dividing them by both B and P expressions" can be interpretated in different ways. To remove any ambiguity I would suggest "by dividing them by the product of B and P expressions"
- Page 8: the rational for dividing (linear scale) SIMPL signals by the product of SB and SP and not e.g. SB+SP, or arithmetic mean of SB and SP, or geometric mean of SB and SP is not explained.
- Page 9: "ROC analysis furthermore demonstrated a significantly improved AUC (0.864) and the cutoff value (0.483) of PRS recovery...", this sentence is probably meant without the "the".
- Page 9, next sentence "appears to be an effect approach", I guess "effective approach" was meant.

Point-by-point response

Response to Reviewer #2

Comment 1: This paper is very clearly written and a very clear essential presentation of the substantially improved method. I would indeed argue that the triple-nanoLUC system presented here advances the method from a low throughput specialized application to a well controlled and broadly applicable, quantitative method in PPI research. The benchmark is thorough and useful, specific examples provide excellent data on PPI inhibition. The assay was performed in 384 plate format and given well defined PPI pairs it would clearly enable a drug screening endeavor! It is an excellent, highly focused systems biology methods-study.
Response to Comment 1: We would like to thank the reviewer for the positive comment.

Comment 2: It is not fully discussed whether cells are lysed or only permeable, and whether the system would work in the lysate as well. This refers to drug treatment. How do we know when to add the drug? In the Ras-Raf experiment induction and addition of the drug happen together for 16h. How do we control for expression level changes upon drug treatment for 16h? For the glue experiments 3h small molecule treatment was used. Can the PPI be induced after permeabilization?
Response to Comment 2: We thank the reviewer for raising these important issues. In the assay, 0.15% NP40 was used. This detergent concentration mainly permeabilizes membranes and should not destroy the major cytoskeleton structure. For PPI inhibitor studies, one of our major concerns is the irreversibility of interaction-induced splicing because once it occurs it can not be counteracted by any inhibitors. Thus, the order of inhibitor treatment and target expression does matter. We use a Tet-on system to control expression and start induction and inhibitor treatment simultaneously. The time lag for protein production guarantees target expression occurs in the presence of inhibitor. The length of treatment can be varied. Although we used 16 hours in the sotorasib experiment, effects can also be achieved in as short as 5 hours although with some reduced signal. In contrast, the situation is different in chemical induced proximity because interaction/splicing does not occur or occurs at very low levels before compound treatment in the cases of rapamycin, RO-5963 and AVR-825. Thus, expression induction can start before or simultaneously with compound treatment. The treatment length can also be varied, with the balance of signal amplitude and minimal side effects caused by compounds.
Even though some PPIs occur under the condition of permeabilization or lysis, protein expression is unlikely to be induced under these conditions. Thus, inhibitors cannot be reliably followed if added solely at this point. However, SIMPL can be modified by expressing two proteins separately followed by mixing the lysates from both expressing cells. Indeed, we successfully tested this approach for KRAS/RBD interaction, which is included in our recent publication (BBRC 734:150792). The behavior of inhibitors can be examined using this alternative SIMPL2 approach like many common *in vitro* assays.
In response to this comment, we modified the text in the Methods section (page 22) to provide clearer descriptions, and addressed the order of induction and inhibitor treatment in the Discussion section (page 17) of the revised manuscript.

Comment 3: Because of the irreversibility of the splicing event, I would assume the complexes are removed from binding equilibrium. Can the authors discuss why then the dose dependent binding inhibition actually works so nicely?

Response to comment 3: This is a very interesting issue. If the reaction occurs in an *in vitro* environment, the irreversibility will drive the reaction in a direction that finally consumes at least one of the interacting proteins. However, in our *in vivo* system, spliced protein undergoes degradation, and nascent unbound proteins are continuously produced. These conditions produce a dynamic equilibrium, or steady state, which provides each species in a relatively constant concentration. Similarly, the presence of an inhibitor produces a steady state with reduced but constant spliced protein, thus creating a set of dynamics apparently resembling inhibitor kinetics. We did not discuss this issue in the manuscript because we think it is out of the scope of the current study. The mathematical models describing SIMPL behavior will, however, be addressed in our future studies.

Comment 4: Intrinsic splicing dependent signals vary depending on the construct. Are there protein constructs where the signal with the respective handle reaches signals so high that they need to be fully excluded from the analysis? This would be the analogy to autoactivation of certain proteins, observed in other types assays.
Response to comment 4: So far, we have not found this kind of outlier in our SIMPL2 assay and thus we have not had to exclude any data. We did choose some proteins which demonstrated very high expression in previous assays and titrated their expression with different doses of doxycycline. Under these conditions, the obtained signals demonstrated a good linearity, suggesting their behaviors still follow the law of mass action.

Comment 5: In the AUC analyses it says what the sensitivity is at the chosen cut off, but not what is the luminescence signal cut off actually.
Response to comment 5: We appreciate the reviewer for pointing out the inappropriate use of the term “cutoff”. A proper description should be “the sensitivity in the condition of 100% specificity”. To make it clearer, we have removed these descriptions in the revised manuscript and instead showed the sensitivities calculated at several different specificity values (including 100% specificity) in a new figure (Fig. EV2E), and outcome of converse calculation: specificities at different sensitivity values (Fig. EV2F). Please find the new description in pages 9-10.

Comment 6: Maybe the global screening strategy "Thus, we conclude that SIMPL2 is well suited for high throughput PPI discovery by screening large open reading frame (ORF) pools fused to IN or IC." is an overstatement, or needs more details. How would you sort out the binders from non-binders in a pooled experiment? The luciferase signal is not connected to DNA or so ...
Response to comment 6: We are sorry for the incorrect statement. Indeed, we meant to claim that SIMPL2 is suitable for assay-based HTS. We agree with the reviewer that in its current state the SIMPL2 assay cannot be applied to screening of pooled libraries. We have corrected the statement (page 16) and modified our tone.

Comment 7: ... of rapid experimental validation which does not bottleneck the discovery process ...Can "bottleneck" be used as a verb like in this sentence?
Response to comment 7: We have changed the word as a noun according (page 17). Thanks for pointing out the mistake.

Response to reviewer #3:

Comment 1: Altogether the authors provide convincing evidence that SIMPL2 is an upgrade compared to SIMPL with higher sensitivity and much easier and cost-effective handling. The

data they use to make their point were generated with well-designed experiments. My main criticisms to the manuscript are the following:
- the repeating tendency of the authors to over emphasize the positive aspects of SIMPL2 while superficially discussing its limitations.
- The lack of evidence that SIMPL2 brings any added value compared to a split-nanoluc (NanoBiT) assay. This one is my main criticism as it considerably limits the general interest for this manuscript.

Response to comment 1: We are grateful to the reviewer for their careful review of our manuscript and comprehensive comments. We have modified the manuscript to try to tone down our overemphasis of the positive aspects of SIMPL2. We also have now included an experimental comparison of SIMPL2 and NanoBiT using three examples which are included in the revised manuscript: rapamycin-induced FRB/FKBP1A interaction (Fig. 1D), KRAS inhibitor (Fig. EV3A) and PROTAC (Fig. EV4B). Please see more detailed descriptions in the following responses.

Comment 2: It is claimed that SIMPL2 demonstrates "an improvement in both sensitivity and specificity over the original SIMPL assay" (abstract and introduction on page 4). However there is only a single direct comparison of SIMPL vs SIMPL2 provided on Fig. 1D. It does suggest improved sensitivity of SIMPL2 but does not provide any evidence of improved specificity. No other data in the manuscript allow making this latter point (a strategy for data transformation is presented on Fig.2 that leads to a more specific identification of hits within the SIMPL2 framework but whether SIMPL2 is more specific than SIMPL has not been addressed).

Response to comment 2: We are glad that the reviewer is convinced about the improved sensitivity of SIMPL2 and appreciate the concerns about specificity.

By definition, specificity should be defined using multiple PPIs. To this end, analyses based on reference sets should provide a clear picture of specificity and are in fact a well accepted method for this purpose. In our assay of reference sets, we introduced an algorithm of data transformation. The result has been better demonstrated using more detailed analyses of the ROC curves, which are included in revised Fig. EV2: better sensitivity was obtained in the transformed data at fixed specificities (Fig. EV2E), and better specificity was obtained at fixed sensitivities (Fig. EV2F). Please find more detailed description in pages 9-10 of revised manuscript. Thus, we are convinced that data transformation can significantly improve both sensitivity and specificity.

In response to the comment, we also compared the ROC curve of a SIMPL2 assay with a SIMPL-ELISA curve from our previous publication, both using the same sets of references. We found that better sensitivity can be achieved with less sacrifice of specificity in SIMPL2. For example, at the point of 100% specificity, SIMPL2 allowed a ~50% sensitivity while only ~33% was achieved in SIMPL-ELISA assay. These data are included as Fig. EV2G (described in page 10) in the revised manuscript. We therefore are convinced that SIMPL2 is more specific than previous version of SIMPL.

Comment 3: It appears to me that a split-nanoluc assay (NanoBiT) would be a better choice than SIMPL2 in most situations: it is an easier setup (two fusion proteins, does not require cell permeabilization and apart from furimazine no additional reagent), is more sensitive (for the rapamycin7FRB/FKBP system Promega shows an EC50 of 1.7 nm for rapamycin measured with NanoBiT vs. 5.5 nm measured with SIMPL2 in the present study), and has the huge advantage of being reversible (and hence allows studying dynamic PPI, also with wash out and/or competition experiments). Can the authors provide experimental evidence that there any reason/specific situation to use SIMPL2 rather than NanoBiT?

Response to comment 3: To address this comment, we experimentally compared the performance of SIMPL2 and NanoBiT in three examples as follow.

1. Rapamycin induced FRB/FKBP1A interaction (revised Fig. 1D). Here, the two assays presented almost identical EC50 values: 5.0 nM for SIMPL2 and 5.2 nM for NanoBiT, which are similar to the values published by Promega (Fig. 6a in ACS Chem. Biol. 11:400): ~6.5 nM. Thus, SIMPL2 did not show less sensitivity than NanoBiT.
2. KRAS inhibitor (revised Fig. EV3A). We tested BI2865, a new pan-KRAS inhibitor (Nature 619: 160–166) in both SIMPL2 and NanoBiT systems. The obtained IC50 values are comparable, 37 nM for SIMPL2 and 24 nM for NanoBiT. These data together with the results in Fig. 3 suggest SIMPL2 is capable of characterizing PPI inhibitors and can be a good alternative to other methods such as NanoBiT. We speculate that SIMPL2 might be less powerful for inhibitors of PPIs with fast association kinetics due to its irreversibility. However, this speculation needs to be experimentally verified in the future.
3. PROTAC (revised Fig. EV4B). ARV-825 induced BRD4/CRBN proximity was also assayed in NanoBiT, in which the obtained signal was low, signal/background ratio (less than 3) was low and z' was negative. In contrast, the signals obtained from SIMPL2 were more than two orders of magnitude higher than NanoBiT, producing about 8 times signal/background ratio and decent z' (0.63). This example suggests a better performance of SIMPL2 in weak PPIs.

In our previous publication (Nature Communications 11:2440), we demonstrated that SIMPL created better sensitivity than many other methods, in agreement with the conclusion that SIMPL may play a better role in characterizing weak PPIs and related modulators, a conclusion drawn from the above supplement experiments. We are fully aware of the shortcomings of SIMPL and addressed this issue in our previous publication. More detailed discussion has been added in the revised manuscript in response to this comment (page 17).
In addition, practically SIMPL2 is not more difficult in experimental setup than NanoBiT because it involves only one step addition of all required components.

Comment 4: The general tone and argumentation of the manuscript presents SIMPL2 almost as the panacea when studying PPI with all other available assays described as replete with limitations. I acknowledge that the authors are excited about their new method but they should try not be too carried out by enthusiasm and thereby forget to guide the non-seasoned reader in avoiding potential pitfalls attached to the assay.
Response to comment 4: We are sorry for giving the reviewer this impression. We have modified the manuscript and tried to be more conservative in our assessments/descriptions.

Comment 5: The assay suffers from its non-reversibility combined with the background splicing activity (shown on Fig. EV2) of the intein fragments.
Response to comment 5: As addressed in Response to comment 3, the irreversibility contributes both advantages and disadvantages to SIMPL. Some disadvantages can be overcome as addressed in the following responses.

Comment 6: This imposes limitations that are not clearly stated:
a. SIMPL2 can be used to test PPI modulators but only if the modulator is applied to the cells before the tested proteins are expressed (otherwise irreversible splicing will have already occurred). This means the suitable setups are virtually only transient transfections or inducible stable cell lines.
Response to comment 6: We used inducible stable cell lines in SIMPL2. We don't think this is a disadvantage or problem.

Comment 7: b. Non-reversibility + low, but detectable, background protein splicing activity also implies that tagging constitutively expressed proteins (e.g. through genome editing or even transient expression over a long time) is most probably not possible: this is not suitable in any case for studying PPI modulators (see 3.a.) and will lead to huge background signal for any pair

of intein-tagged proteins (constitutive expression over days will necessarily lead to the accumulation of intein-mediated ligated products over time due to the background activity).
Response to comment 7: Strictly, characterization of inhibitors targeting many PPIs using SIMPL is not possible in constitutively expressing cells. These don't include some conditional PPIs or chemical induced proximity. However, this problem most probably can be overcome by induced expression.

Comment 8: c. It appears (Fig. EV2C & D) that there is a significant number of PRS for which the signal of the negative control (SB or SP) is higher than the SIMPL2 signal, which means that for those pair of proteins with validated binary interaction, coupling one of the two with an IC or IN handle yields a higher signal than when measuring the SIMPL2 constructs. I am aware that with the data transformation strategy presented on Fig. 2, the authors provide a strategy to define a cutoff that will lead to the identification of real positive hits most of the time. However this is feasible in a HTS set up but for those testing a single pair of proteins this strategy will not be applicable and it means that performing both IC and IN handle controls is critical to ensure the SIMPL2 signals are specific. This is not enough emphasized in the conclusion of the article.
Response to comment 8: This is an insightful consideration. We are in a process of developing a framework using a minimal number of protein pairs as universal negative standards for cutoff value determination, which should be published in the future.

Comment 9: d. Looking at the data it seems to me that the expression levels of the fusion proteins must be kept under a certain threshold: on Fig. 2C, F, I it looks like above a SBP (log) of approx. 6.5 the background signal is just too high to allow identify any real interaction. Can the authors comment on this, does this threshold clearly corresponds to an overexpression or does it mean that above a certain physiological expression level SIMPL2 is just not applicable?
Response to comment 9: This is an interesting observation. However, we are not sure if this observation is statistically significant: they are in a very small fraction. Moreover, the point with the highest SBP value is also accompanied with a positive SIMPL2 signal after transformation. Therefore, we don't want to make any speculation at this stage. But we do think it is worthwhile to characterize more PPIs with high expression in SIMPL2 system.

Comment 10: Fig. 3A: in contrast to what the main text claims there is an inhibition (approx. 40-50%) observed at high sotorasib concentrations when the negative control HRAS/RBD and EGF/SHC1 cells were tested. This needs a comment.
Response to comment 10: It should be clarified that the inhibition occurred only in EGFR/SHC1 but not HRAS/RBD. This off-target effect can be brought about by many reasons. One possible reason is the interference of endogenous signaling pathways involved in the expression of target protein expression. This is a common shortcoming in cell-based assays, including NanoBiT. Usually, it can be easily distinguished due to its high concentration. Counter screening and complement with other assays, especially *in vitro* methods, can also help exclude this effect. The possible off-target effects in cell-based assay were addressed in Discussion (pag 17).

Comment 11: the rational for testing WT and L858R variant of EGFR is not explained. Was it expected that the two tested compounds would inhibit the oncogenic variant more efficiently?
Response to comment 11: Yes, we expected to see more significant inhibition in L858R mutant as previously reported. We have modified the manuscript to make this clearer (pag 17).

Comment 12: For all the IC50 measured, how do they compare with previously published data?
Response to comment 12: The comparisons have been included in the revised manuscript (line 6-7 in page 11 and line 1-2 in page 12). They are well in line with reported inhibition profiles.

Though it should be noted that IC50 can vary significantly depending on the assay used.

Comment 13: In the abstract, for the sake of precision please write : "... for detecting binary PPI via..." instead of : "... for detecting PPI via..."
Response to comment 13: Thank you for the comment. The change has been made in the revised manuscript (page 2).

Comment 14: Page 4: "providing a means to help meet the current demand for new therapeutic approaches to treat serious disease." This far-fetched statement is really not needed, the method is nice enough by itself that it does not need to be pimped with the extraordinary claim that it will help cure serious disease. It is a nice addition indeed to the toolbox of methods for studying PPIs, but how it will be used for in the future and what it will help to achieve is anyone's guess.
Response to comment 14: We have deleted this claim in the revised manuscript according to this comment.

Comment 15: Page 7. In the sentence "However, if the proteins under study are sufficiently stable, even trace levels of background, non-specifically spliced product could accumulate..." I guess the authors meant "...even trace with levels of background..."
Response to comment 15:
Response to comment 15: We have changed to a cleared expression in the revised manuscript (line 4, paragraph 3 in page 7).

Comment 16: The explanations for the PRS and RRS sets are limited to the sentence "We next used reference PPIs to evaluate the performance of the SIMPL2 system. hsPRS-v2 (n=60) was used as the positive reference set (PRS) in this study30. The random reference set (RRS) contains 80 protein pairs with baits and preys selected from hsPRS-v1" on page 7. Please consider changing the wording (this study: does it refer to the present study or to ref. 30?) add more explanations for the non-seasoned reader: without knowing Ref. 30, the reader has no idea what are hsPRS-v2 and hsPRS-v1 and what was the rational to construct the RRS set from v1 rather than v2. At the present these few lines are too quick on the explanations making it hard to follow.
Response to comment 16: We have modified the revised manuscript according to this comment. More details of hsPRS-v1 and hsPRS-v2 were provided.

Comment 17: Page 8: the wording "by dividing them by both B and P expressions" can be interpretated in different ways. To remove any ambiguity I would suggest "by dividing them by the product of B and P expressions"
Response to comment 17: We have changed in the revised manuscript according to this comment (page 8).

Comment 18: Page 8: the rational for dividing (linear scale) SIMPL signals by the product of SB and SP and not e.g. SB+SP, or arithmetic mean of SB and SP, or geometric mean of SB and SP is not explained.
Response to comment 18: This is indeed like two-dimensional normalization. Since the binding process follows the law of mass action (second-order), $[BP] = \frac{1}{K_D}[B][P]$, expression product should be used rather than other measures. More detail about the mathematics in the kinetics model of SIMPL2 will be addressed in our future research.

Comment 19: Page 9: "ROC analysis furthermore demonstrated a significantly improved AUC

(0.864) and the cutoff value (0.483) of PRS recovery...", this sentence is probably meant without the "the".
Response to comment 19: We have changed in the revised manuscript according to this comment.

Comment20: Page 9, next sentence "appears to be an effect approach", I guess "effective approach" was meant.
Response to comment 20: We have changed in the revised manuscript according to this comment.

13th Nov 2024

Manuscript Number: MSB-2024-12338R
Title: A Split Intein and Split Luciferase-Coupled System for Detecting Protein-Protein Interactions

Dear Prof Stagljar,

Thank you for the submission of your revised manuscript to Molecular Systems Biology. We have now received the enclosed reports from the referees that were asked to re-assess it. As you will see the reviewers are now globally supportive and I am pleased to inform you that we will be able to accept your manuscript pending the following final amendments:

1) Please rename "Conflict of Interest" to "Disclosure and competing interests statement". We updated our journal's competing interests policy in January 2022 and request authors to consider both actual and perceived competing interests. Please review the policy https://www.embopress.org/competing-interests and update your competing interests if necessary.
2) Author contributions: Please remove it from the manuscript and specify author contributions in our submission system. CRediT has replaced the traditional author contributions section because it offers a systematic machine-readable author contributions format that allows for more effective research assessment. You are encouraged to use the free text boxes beneath each contributing author's name to add specific details on the author's contribution. More information is available in our guide to authors:
https://www.embopress.org/page/journal/17574684/authorguide#authorshipguidelines
3) In the Methods, please take care of the following:
- Cell lines: Please also be sure to include a sentence in the Methods as to whether or not the cell lines were recently authenticated and tested for mycoplasma contamination. Please also be sure to update the Author Checklist with this information and where it can be found in the manuscript.
- Please ensure that a statement on whether or not blinding was done is included in the Methods even if no blinding was done. Please also be sure to update the Author Checklist with this information and where it can be found in the manuscript.
- Please remove the Reagents and Tools table from the manuscript as you have already provided as a separate .docx file.
4) Please place individual sections of the manuscript in the following order: Title page - Abstract & Keywords - Introduction - Results - Discussion - Methods - Data Availability - Acknowledgements - Disclosure and Competing Interests Statement - References - Figure Legends - Expanded View Figure Legends.
5) For the figures and figure legends, please take care of the following:
- Please note that the box plots need to be defined in terms of minima, maxima, centre, bounds of box and whiskers, and percentile in the legends of figures 2A, D, G.
- Please note that information related to n is missing in the legends of figures 2A, D, G.
- Although 'n' is provided, please describe the nature of entity for 'n' in the legends of figures 1B; 3A-D; 4A, B; EV1 B, C; EV3 A, EV4 B.
6) Appendix file: Please include a title page for the Appendix and include a Table of Contents with page numbers.
7) Synopsis:
- Synopsis text: Please remove the synopsis from the main manuscript file and upload it as a separate file.
- Please check your synopsis text and image before submission with your revised manuscript. Please be aware that in the proof stage minor corrections only are allowed (e.g., typos).
8) Source Data: Please ensure that a completed Source Data checklist is uploaded as a Related Manuscript File. Source Data should be organized as a single source data file (zipped) per figure for main figures (all EV and/or Appendix figure Source Data can be included in a single folder), with the panels clearly visible in the folder structure instead of a single excel file for all Source Data. e.g. all the Source data files for figure 1 need to be saved in a single folder and this needs to be zipped and then uploaded as "SD figure 1.zip" file.
9) As part of the EMBO Publications transparent editorial process initiative (see our policy here: https://www.embopress.org/transparent-process#Review_Process), Molecular Systems Biology will publish online a Peer Review File (PRF) to accompany accepted manuscripts. This file will be published in conjunction with your paper and will include the anonymous referee reports, your point-by-point response and all pertinent correspondence relating to the manuscript. Let us know whether you agree with the publication of the PRF and as here, if you want to remove or not any figures from it prior to publication. Please note that the Authors checklist will be published at the end of the PRF.
10) Please provide a point-by-point letter INCLUDING my comments and your detailed responses (as Word file).

I look forward to reading a new revised version of your manuscript as soon as possible.

Yours sincerely,

Poonam Bheda, PhD
Scientific Editor
Molecular Systems Biology

Reviewer #2:

Revised version Yao et al. " A Split Intein and Split Luciferase-Coupled System for Detecting Protein-Protein Interactions"
While I liked the original submission a lot already, the authors put substantial effort into revising, answering open questions and improving the manusript. I think it is a clear and to the point presentation of a very powerful (in cell) PPI method with great potential for HTP analyses. I would like to congratulate the authors to their work and suggest to go ahead.

Reviewer #3:

I wish to congratulate the authors for excellent revisions to their manuscript. They have now addressed the majority of my and the other reviewer's concerns successfully. I am convinced that the new Simpl2 method reported here will be helpful for numerous researchers studying protein-protein interactions. I fully support the publication of the revised manuscript.

All editorial and formatting issues were resolved by the authors.

29th Nov 2024
Manuscript number: MSB-2024-12338RR
Title: A Split Intein and Split Luciferase-Coupled System for Detecting Protein-Protein Interactions

Dear Prof Stagljar,

Congratulations on an excellent manuscript, I am pleased to inform you that your manuscript has been accepted for publication in Molecular Systems Biology. Thank you for your comprehensive response to referee concerns. It has been a pleasure to work with you to get this to the acceptance stage.

Your manuscript will be processed for publication by EMBO Press. It will be copy edited and you will receive page proofs prior to publication. Please note that you will be contacted by Springer Nature Author Services to complete licensing and payment information.

You may qualify for financial assistance for your publication charges - either via a Springer Nature fully open access agreement or an EMBO initiative. Check your eligibility: https://www.embopress.org/page/journal/17444292/authorguide#chargesguide

Should you be planning a Press Release on your article, please get in contact with embo_production@springernature.com as early as possible in order to coordinate publication and release dates.

If you have any questions, please do not hesitate to contact the Editorial Office. Thank you for your contribution to Molecular Systems Biology.

Yours sincerely,

Sincerely,

Poonam Bheda, PhD
Scientific Editor
Molecular Systems Biology

>>> Please note that it is Molecular Systems Biology policy for the transcript of the editorial process (containing referee reports and your response letter) to be published as an online supplement to each paper. If you do NOT want this, you will need to inform the Editorial Office via email immediately. More information is available here: https://www.embopress.org/transparent-process#Review_Process

EMBO Press Author Checklist

Corresponding Author Name: Igor Stagljar, Zhong Yao	
Journal Submitted to: Molecular Systems Biology	
Manuscript Number: MSB-2024-12338	

USEFUL LINKS FOR COMPLETING THIS FORM
The EMBO Journal - Author Guidelines
EMBO Reports - Author Guidelines
Molecular Systems Biology - Author Guidelines
EMBO Molecular Medicine - Author Guidelines

Reporting Checklist for Life Science Articles (updated January
This checklist is adapted from Materials Design Analysis Reporting (MDAR) Checklist for Authors. MDAR establishes a minimum set of requirements in transparent reporting in the life sciences (see Statement of Task: 10.31222/osf.io/9sm4x). Please follow the journal's guidelines in preparing your
Please note that a copy of this checklist will be published alongside your article.

Abridged guidelines for figures
1. Data
The data shown in figures should satisfy the following conditions:
- the data were obtained and processed according to the field's best practice and are presented to reflect the results of the experiments in an accurate and unbiased manner.
- ideally, figure panels should include only measurements that are directly comparable to each other and obtained with the same assay.
- plots include clearly labeled error bars for independent experiments and sample sizes. Unless justified, error bars should not be shown for technical
- if n<5, the individual data points from each experiment should be plotted. Any statistical test employed should be justified.
- Source Data should be included to report the data underlying figures according to the guidelines set out in the authorship guidelines on Data

2. Captions
Each figure caption should contain the following information, for each panel where they are relevant:
- a specification of the experimental system investigated (eg cell line, species name).
- the assay(s) and method(s) used to carry out the reported observations and measurements.
- an explicit mention of the biological and chemical entity(ies) that are being measured.
- an explicit mention of the biological and chemical entity(ies) that are altered/varied/perturbed in a controlled manner.
- the exact sample size (n) for each experimental group/condition, given as a number, not a range;
- a description of the sample collection allowing the reader to understand whether the samples represent technical or biological replicates (including how many animals, litters, cultures, etc.).
- a statement of how many times the experiment shown was independently replicated in the laboratory.
- definitions of statistical methods and measures:
  - common tests, such as t-test (please specify whether paired vs. unpaired), simple χ2 tests, Wilcoxon and Mann-Whitney tests, can be unambiguously identified by name only, but more complex techniques should be described in the methods section;
  - are tests one-sided or two-sided?
  - are there adjustments for multiple comparisons?
  - exact statistical test results, e.g., P values = x but not P values < x;
  - definition of 'center values' as median or average;
  - definition of error bars as s.d. or s.e.m.

Please complete ALL of the questions below.
Select "Not Applicable" only when the requested information is not relevant for your study.

Materials

Newly Created Materials	Information included in the manuscript?	In which section is the information available? (Reagents and Tools Table, Materials and Methods, Figures, Data Availability Section)
New materials and reagents need to be available; do any restrictions apply?	Yes	Methods, Fig.EV1, Appendix Fig.S1

Antibodies	Information included in the manuscript?	In which section is the information available? (Reagents and Tools Table, Materials and Methods, Figures, Data Availability Section)
For **antibodies** provide the following information:<br>- Commercial antibodies: RRID (if possible) or supplier name, catalogue number and or/clone number<br>- Non-commercial: RRID or citation	Yes	Reagents and Tools Table

DNA and RNA sequences	Information included in the manuscript?	In which section is the information available? (Reagents and Tools Table, Materials and Methods, Figures, Data Availability Section)
**Short novel DNA or RNA including primers, probes**: provide the sequences.	Yes	Methods

Cell materials	Information included in the manuscript?	In which section is the information available? (Reagents and Tools Table, Materials and Methods, Figures, Data Availability Section)
**Cell lines:** Provide species information, strain. Provide accession number in repository **OR** supplier name, catalog number, clone number, and/**OR** RRID.	Yes	Reagents and Tools Table, and Methods
**Primary cultures:** Provide species, strain, sex of origin, genetic modification status.	Not Applicable	
Report if the cell lines were recently **authenticated** (e.g., by STR profiling) and tested for mycoplasma contamination.	Yes	The cells used in this study (HEK293T) were not recently authenticated or tested for mycoplasma contamination. Please check Methods.

Experimental animals	Information included in the manuscript?	In which section is the information available? (Reagents and Tools Table, Materials and Methods, Figures, Data Availability Section)
**Laboratory animals or Model organisms:** Provide species, strain, sex, age, genetic modification status. Provide accession number in repository **OR** supplier name, catalog number, clone number, **OR** RRID.	Not Applicable	
**Animal observed in or captured from the field:** Provide species, sex, and age where possible.	Not Applicable	
Please detail **housing and husbandry conditions**.	Not Applicable	

Plants and microbes	Information included in the manuscript?	In which section is the information available? (Reagents and Tools Table, Materials and Methods, Figures, Data Availability Section)
**Plants:** provide species and strain, ecotype and cultivar where relevant, unique accession number if available, and source (including location for collected wild specimens).	Not Applicable	
**Microbes:** provide species and strain, unique accession number if available, and source.	Not Applicable	

Human research participants	Information included in the manuscript?	In which section is the information available? (Reagents and Tools Table, Materials and Methods, Figures, Data Availability Section)
If collected and within the bounds of privacy constraints report on age, sex and gender or ethnicity for all study participants.	Not Applicable	

Core facilities	Information included in the manuscript?	In which section is the information available? (Reagents and Tools Table, Materials and Methods, Figures, Data Availability Section)
If your work benefited from core facilities, was their service mentioned in the acknowledgments section?	Not Applicable	

Design

Study protocol	Information included in the manuscript?	In which section is the information available? (Reagents and Tools Table, Materials and Methods, Figures, Data Availability Section)
If study protocol has been **pre-registered, provide DOI in the manuscript**. For clinical trials, provide the trial registration number **OR** cite DOI.	Not Applicable	
Report the **clinical trial registration number** (at ClinicalTrials.gov or equivalent), where applicable.	Not Applicable	

Laboratory protocol	Information included in the manuscript?	In which section is the information available? (Reagents and Tools Table, Materials and Methods, Figures, Data Availability Section)
Provide DOI OR other citation details if **external detailed step-by-step protocols** are available.	Not Applicable	

Experimental study design and statistics	Information included in the manuscript?	In which section is the information available? (Reagents and Tools Table, Materials and Methods, Figures, Data Availability Section)
Include a statement about **sample size** estimate even if no statistical methods were used.	Yes	Results
Were any steps taken to minimize the effects of subjective bias when allocating animals/samples to treatment (e.g. **randomization procedure**)? If yes, have they been described?	Not Applicable	
Include a statement about **blinding** even if no blinding was done.	Yes	Statistics section in Methods
Describe **inclusion/exclusion criteria** if samples or animals were excluded from the analysis. Were the criteria pre-established?<br><br>If sample or data points were omitted from analysis, report if this was due to attrition or intentional exclusion and provide justification.	Not Applicable	
For every figure, are **statistical tests** justified as appropriate? Do the data meet the assumptions of the tests (e.g., normal distribution)? Describe any methods used to assess it. Is there an estimate of variation within each group of data? Is the variance similar between the groups that are being statistically compared?	Not Applicable	

Sample definition and in-laboratory replication	Information included in the manuscript?	In which section is the information available? (Reagents and Tools Table, Materials and Methods, Figures, Data Availability Section)
In the figure legends: state number of times the experiment was **replicated** in laboratory.	Yes	Figures
In the figure legends: define whether data describe **technical or biological replicates**.	Yes	Figures

Ethics

Ethics	Information included in the manuscript?	In which section is the information available? (Reagents and Tools Table, Materials and Methods, Figures, Data Availability Section)
Studies involving **human participants**: State details of **authority granting ethics approval** (IRB or equivalent committee(s), provide reference number for approval.	Not Applicable	
Studies involving **human participants**: Include a statement confirming that **informed consent** was obtained from all subjects and that the experiments conformed to the principles set out in the WMA Declaration of Helsinki and the Department of Health and Human Services Belmont Report.	Not Applicable	
Studies involving **human participants:** For publication of **patient photos**, include a statement confirming that consent to publish was obtained.	Not Applicable	
Studies involving experimental **animals**: State details of **authority granting ethics approval** (IRB or equivalent committee(s), provide reference number for approval. Include a statement of compliance with ethical regulations.	Not Applicable	
Studies involving **specimen and field samples:** State if relevant **permits** obtained, provide details of authority approving study; if none were required, explain why.	Not Applicable	

Dual Use Research of Concern (DURC)	Information included in the manuscript?	In which section is the information available? (Reagents and Tools Table, Materials and Methods, Figures, Data Availability Section)
Could your study fall under dual use research restrictions? Please check biosecurity documents and list of **select agents and toxins** (CDC): https://www.selectagents.gov/sat/list.htm	Not Applicable	
If you used a select agent, is the security level of the lab appropriate and reported in the manuscript?	Not Applicable	
If a study is subject to dual use research of concern regulations, is the name of the **authority granting approval and reference number** for the regulatory approval provided in the manuscript?	Not Applicable	

Reporting

The MDAR framework recommends adoption of discipline-specific guidelines, established and endorsed through community initiatives. Journals have their own policy about requiring specific guidelines and recommendations to complement MDAR.

Adherence to community standards	Information included in the manuscript?	In which section is the information available? (Reagents and Tools Table, Materials and Methods, Figures, Data Availability Section)
State if relevant guidelines or checklists (e.g., **ICMJE, MIBBI, ARRIVE, PRISMA**) have been followed or provided.	Not Applicable	
For **tumor marker prognostic studies,** we recommend that you follow the **REMARK** reporting guidelines (see link list at top right). See author guidelines, under 'Reporting Guidelines'. Please confirm you have followed these guidelines.	Not Applicable	
For **phase II and III randomized controlled trials**, please refer to the **CONSORT** flow diagram (see link list at top right) and submit the CONSORT checklist (see link list at top right) with your submission. See author guidelines, under 'Reporting Guidelines'. Please confirm you have submitted this list.	Not Applicable	

Data Availability

Data availability	Information included in the manuscript?	In which section is the information available? (Reagents and Tools Table, Materials and Methods, Figures, Data Availability Section)
Have **primary datasets** been deposited according to the journal's guidelines (see 'Data Deposition' section) and the respective accession numbers provided in the Data Availability Section?	Not Applicable	
Were **human clinical and genomic datasets** deposited in a public access-controlled repository in accordance to ethical obligations to the patients and to the applicable consent agreement?	Not Applicable	
Are **computational models** that are central and integral to a study available without restrictions in a machine-readable form? Were the relevant accession numbers or links provided?	Not Applicable	
If publicly available data were reused, provide the respective **data citations in the reference list**.	Yes	Results and References
